# Neurotransmitter signaling in the pathophysiology of microglia

**DOI:** 10.3389/fncel.2013.00049

**Published:** 2013-04-19

**Authors:** María Domercq, Nuria Vázquez-Villoldo, Carlos Matute

**Affiliations:** ^1^Departamento de Neurociencias, Universidad del País Vasco-UPV/EHULeioa, Spain; ^2^Achucarro Basque Center for Neuroscience-UPV/EHUZamudio, Spain; ^3^Instituto de Salud Carlos III, Centro de Investigación Biomédica en Red de Enfermedades NeurodegenerativasLeioa, Spain

**Keywords:** microglia, ATP, glutamate, purinergic and glutamatergic receptors

## Abstract

Microglial cells are the resident immune cells of the central nervous system. In the resting state, microglia are highly dynamic and control the environment by rapidly extending and retracting motile processes. Microglia are closely associated with astrocytes and neurons, particularly at the synapses, and more recent data indicate that neurotransmission plays a role in regulating the morphology and function of surveying/resting microglia, as they are endowed with receptors for most known neurotransmitters. In particular, microglia express receptors for ATP and glutamate, which regulate microglial motility. After local damage, the release of ATP induces microgliosis and activated microglial cells migrate to the site of injury, proliferate, and phagocytose cells, and cellular compartments. However, excessive activation of microglia could contribute to the progression of chronic neurodegenerative diseases, though the underlying mechanisms are still unclear. Microglia have the capacity to release a large number of substances that can be detrimental to the surrounding neurons, including glutamate, ATP, and reactive oxygen species. However, how altered neurotransmission following acute insults or chronic neurodegenerative conditions modulates microglial functions is still poorly understood. This review summarizes the relevant data regarding the role of neurotransmitter receptors in microglial physiology and pathology.

## Introduction

Microglial cells constitute the resident immune cell population of the mammalian central nervous system (CNS). Postnatally, microglia are present in all regions of the CNS in a non-overlapping territorial manner and comprise a large proportion of the total cellular makeup of the CNS, which is estimated to be as high as 12% (Lawson et al., 1990). Similar to macrophages, their peripheral counterparts, microglia display remarkable ranges of morphology and activity depending, in part, on the state of the surrounding tissue (Lynch, 2009; Ransohoff and Perry, 2009). “Resting” microglia are not functionally silent cells, but extremely dynamic *in vivo*, perpetually changing their morphology by extending and retracting highly motile processes on a time scale of minutes (Davalos et al., 2005; Nimmerjahn et al., 2005). In response to local damage (few micron lesions), microglial processes rapidly and automatically converge on the site of injury without cell body movement. The microglial branching response mediated by ATP release aims to shield and/or scavenge the affected side. In addition, part of the dynamic motility of surveying microglial processes *in vivo* is directed toward synapses, suggesting that microglia vigilantly monitor and respond to the functional status of synapses (Wake et al., 2009). In addition, microglia have been reported to be capable of sensing defunct synapses and phagocytose them in normal brain (Wake et al., 2009; Tremblay et al., 2010). Synaptic pruning by microglia is essential during development for the remodeling of synaptic circuits [Paolicelli et al., 2011; see also the reviews by Tremblay (2011) and Wake et al. (2013)]. Microglia also efficiently phagocytose apoptotic neurons in the neurogenic niche (Sierra et al., 2010).

In addition to its functions in normal brain, microglia are involved in most, if not all, known CNS pathologies. More than a decade ago, Georg Kreutzberg coined the term “microglial sensor of pathology” (Kreutzberg, 1996), which captures the essence of microglial cell function. Microglia are the brain's intrinsic immune cells and serve as damage sensors within the brain. Any type of injury or pathological process leads to the activation of these cells from their surveillant/resting state. In response to injury, microglia change their highly branched and ramified morphology by retracting their processes and taking on an ameboid appearance. Activated microglial cells can then migrate to the site of injury, proliferate, and release substances that affect pathological processes. These substances include pro-inflammatory cytokines, such as tumor necrosis factor (TNF)-α, and interleukin (IL)-6 or IL-12, which signal the invading T lymphocytes.

Multiple signals converge on microglial cells to actively maintain or alter their functional state and orchestrate the specific repertoire of microglial functions. Transitions between surveillance and activated states are triggered when microglia perceive a sudden appearance, abnormal concentration, or unusual molecular format of certain factors (Hanisch and Kettenmann, 2007). This review focuses on the role of neurotransmitter receptors, particular ATP and glutamate receptors, in the control of microglial physiology and pathology. For the role of other receptors or channels, see these other excellent reviews (Pocock and Kettenmann, 2007; Kettenmann et al., 2011).

## ATP receptors

### Expression of ATP receptors in microglia

Purines and pyrimidines act as widespread extracellular signaling molecules. The physiological effects of purines and pyrimidines are mediated through an extended family of purinoceptors activated by adenosine, classified as P1 receptors, or by ATP, classified as P2 receptors (Ralevic and Burnstock, 1998; North, 2002). Purinergic receptors are expressed in the majority of living cells and are particularly abundant in glia (Pocock and Kettenmann, 2007; Kettenmann et al., 2011). ATP activates a family of metabotropic P2Y, P2Y_1_, P2Y_2_, P2Y_4_, P2Y_6_, P2Y_11_, P2Y_12_, P2Y_13_, P2Y_14_, and ionotropic P2X1-7 receptors. Extracellular ATP is degraded to adenosine by ectonucleotidases, such as CD39 and CD73, which are known to be present in microglial cells (Braun et al., 2000) and adenosine activates G protein-coupled adenosine receptors A_1_, A_2A_, A_2B_, and A_3_. The A_1_ and A_3_ receptors can inhibit adenylyl cyclase or activate phospholipase C, whereas A_2A_ and A_2B_ receptors activate cyclic AMP production (Fredholm et al., 2001). Collectively, the actions of ATP and its degradation products produce responses that last from milliseconds to minutes, and even longer time scales through changes in gene regulation via second messengers (Khakh and North, 2012). P2X receptors are non-selective cation channels with high Ca^2+^ permeability that carry a depolarizing current under standard physiological conditions. In some cells, P2X channels are also significantly permeable to anions, such as the full-length P2X5 receptor (P2X5R), which is permeable to Cl^−^ (North, 2002). Functional homomeric P2X1R and P2X3Rs have fast desensitization properties. The other receptor types have slow desensitization properties, except P2X7R, which does not desensitize (Khakh and North, 2012). After prolonged activation, P2X7Rs open a large pore, causing cytolytic cell death (Surprenant et al., 1996). Signaling diversity is increased by the broad range of ATP sensitivities exhibited by ATP receptors, ranging from nanomoles in the case of P2Y receptors to hundreds of micromoles for P2X7Rs (North, 2002). Thus, ATP receptors respond over remarkably broad spatiotemporal scales, making ATP signaling highly dynamic.

P2X-mediated currents were identified in microglial cultures prepared from human and rodent brains more than two decades ago (Walz et al., 1993). Patch-clamp recordings have shown that cultured microglial cells respond to extracellular ATP (100 μM) with the activation of a transient inward non-selective cationic current, which is followed, in some cases, by an outward K^+^ current (Walz et al., 1993; Nörenberg et al., 1994; McLarnon et al., 1999). These results were recently corroborated and extended in acute slices. Thus, ATP triggers a non-selective cationic inward current in association with the activation of P2X7Rs, and an outward K^+^ current associated with the activation of P2Y6 and P2Y12 metabotropic receptors (Boucsein et al., 2003; Avignone et al., 2008). Importantly, these studies describe diverse electrophysiological properties for microglial cell types, one subtype with a lower resting membrane potential (between −50 mV and −60 mV) and another subtype with a higher membrane potential (−20 to −30 mV), and on the basis of different responses to ATP. These differences may be associated with the different functional roles of microglia.

Immunohistochemical studies have shown the expression of low levels of P2X4 (Ulmann et al., 2008) and P2X7 (Matute et al., 2007) and high levels of P2Y12 (Haynes et al., 2006) in microglia in the adult normal brain. The expression of purinergic receptors changes during development (Xiang and Burnstock, 2005). Thus, at embryonic day 16, the majority of microglial cells express P2X1 and P2X4 subunits, whereas only 30% of these cells express P2X7. From postnatal day 7, P2X4-positive microglia locate preferentially around blood vessels. At postnatal P30, the cells expressing P2X1 virtually disappear, the P2X7-positive cells are distributed widely through the forebrain, whereas cells bearing P2X4Rs are mainly localized around blood vessels and lining the subarachnoid space (Xiang and Burnstock, 2005). Constitutive expression of P2X4 in pervivascular cells was also described in the adult spinal cord (Guo and Schluesener, 2005). Pervivascular P2X4^+^ cells are ED1^+^/OX42^+^, indicating that correspond to infiltrating monocytes/microglia, but not to lymphocytes.

From postnatal day 7, many microglial cells with P2X4 receptor-immunoreactivity were seen around the blood vessels. At postnatal day 30, microglial cells with P2X1 receptor-immunoreactivity disappeared and the cells with P2X4 receptor-immunoreactivity were mainly localized around blood vessels and lining the subarachnoid space. From postnatal day 30, the microglial cells with P2X7 receptor-immunoreactivity were found to be distributed widely in the forebrain. Cells with P2X7 receptor-immunoreactivity from P30 were not labeled by ED1, but some were labeled by isolectin B4. The expression of P2X1, P2X4, and P2X7 receptor mRNA and protein on primary cultures of rat microglial cells and on the N9 microglial cell line was demonstrated with immunocytochemistry and RT-PCR. This is the first report that the P2X1 receptor is expressed on microglial cells, at least in early development, before postnatal day 30.

Regarding adenosine receptors, *in vitro* functional studies have identified the expression of all P1 adenosine receptors, A_1_, A_2A_, A_2B_, and A_3_ receptors in microglia (Haskó et al., 2005; Abbracchio and Ceruti, 2007). *In vivo*, the A_2A_ receptor appears to be expressed only in activated microglia after systemic lipopolysaccharide (LPS) injection (Orr et al., 2009). In contrast, adult healthy brains express relatively higher levels of A_1_ and A_3_ receptors (Koizumi et al., 2013).

### Functions of ATP receptors in microglia

**Figure 1:**
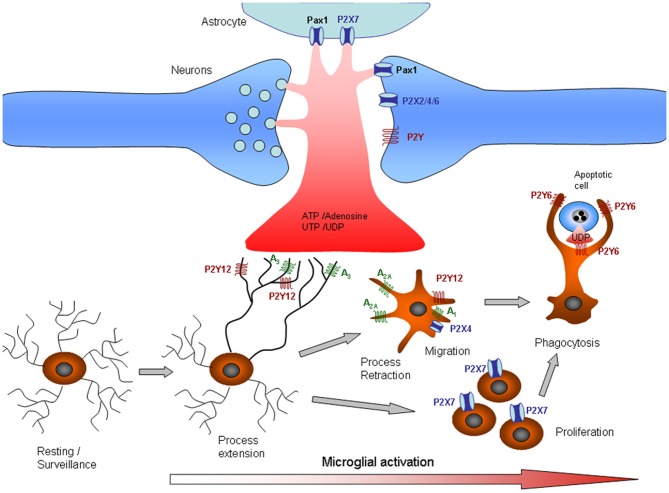
**Purinergic signaling in microglia.** Release or leakage of nucleotides/nucleosides from injured neurons, astrocytes, or microglial cells induces phenotypic alterations in microglia. Microglial processes exhibit constitutive motility, which is dependent on ATP signaling. Microglial processes are rapidly recruited to sites of CNS tissue damage by P2Y12 and A_3_ receptor activation. As the damage progresses, microglia undergo progressive changes, including altered expression of cell surface markers and inflammation-related genes, process retraction and the acquisition of an ameboid morphology, cell body migration, and increasing phagocytic ability. The changes in microglial functions are partly associated with changes in purinergic receptors that determine different responses to ATP. Thus, process retraction is mainly due to upregulation of A_2A_ and downregulation of P2Y12 receptors, whereas migration is mediated by A_1_ and P2X4 receptors and proliferation by P2X7 receptors. Phagocytosis signaling is also unmasked by the upregulation of P2Y6, which is activated by the release of UTP by dying cells. See also Table 1.

The initial microglial responses that occur after brain injury and in various neurological diseases are characterized by microglial accumulation in the affected sites of the brain as a result of the migration and proliferation of these cells. The early-phase signal responsible for this accumulation is likely to be transduced by rapidly diffusible factors, such as ATP. Purinergic receptors control several microglial functions, including the motility of their fine processes, migration, cytokine release, and phagocytosis (Figure 1). Low ATP concentrations almost exclusively activate chemotaxis in order to recruit cells at the site of injury or inflammation. When the ATP concentration increases, additional effector functions, such as phagocytosis and cytokine secretion, are also triggered (Table 1).

**Table 1:** **Expression and function of purinergic receptors on microglia**.

**Receptor type**	**Presence**	**Function**	**References**
**P1**			
A_1_	+	Migration/Neuropathic pain/Antiinflammatory properties	Färber et al., 2008; Haselkorn et al., 2010; Luongo et al., 2012
A_2A_	+	Process retraction/Microglial activation	Orr et al., 2009; Yao et al., 2012
A_2B_	+	Anti-inflammatory properties; release of IL-10	Koscsó et al., 2012
A_3_	+	Process extension and migration	Ohsawa et al., 2012
**P2**			
***Ionotropic***			
P2X1	−	–	Cavaliere et al., 2005; Xiang and Burnstock, 2005
P2X2	−	–	Unpublished observation
P2X3	+	–	Unpublished observaton
P2X4	+	Migration/Neuropathic pain	Ohsawa et al., 2007; Beggs et al., 2012
P2X5	?	–	
P2X6	−	–	Cavaliere et al., 2005
P2X7	+	Microglial proliferation/Inflammasome signaling	Rigato et al., 2012; Volonte et al., 2012
***Metabotropic***			
P2Y1	?	Purine release/Activation of a K^+^ current	Boucsein et al., 2003; Ballerini et al., 2005
P2Y2	+	Aβ(1–42) degradation and uptake	Kim et al., 2012
P2Y4	+	Not determined	–
P2Y6	+	Phagocytosis	Koizumi et al., 2007
P2Y11	?	Microglial activation	Brandenburg et al., 2010
P2Y12	+	Process extension/Migration	Haynes et al., 2006
P2Y13	+	Neuropathic pain	Kobayashi et al., 2012
P2Y14	+	Neuropathic pain	Kobayashi et al., 2012

?, Not determined

Movement of the fine microglial processes is controlled primarily through the activation of P2Y_12_ receptors, which are expressed at high levels in microglia in normal brain. However, P2Y_12_ receptors are downregulated in microglia after stroke or activation by LPS (Haynes et al., 2006). Microglial chemotaxis is characterized by cell body movement, as well as process movement, and is mediated through the activation of P2X4 and P2Y12 receptors (Honda et al., 2001; Ohsawa et al., 2007). Haynes et al. (2006) showed that, in P2Y_12_ receptor-null mice undergoing focal laser cortical ablation, the chemotactic response of microglia was markedly impaired in the first 40 min of the observation period compared to wild-type animals. However, when microglia from mutant mice were examined 2 h after injury, the degree of chemotaxis approached that observed in wild-type animals. Moreover, P2Y12 receptor expression on microglia was barely observable 24 h after injury. The loss of P2Y12 expression accompanied microglial transformation from highly ramified to an amoeboid state. These observations indicate that P2Y12 receptors are involved in the early, rather than late, responses of microglia to injury (Haynes et al., 2006). In contrast, P2X4 has been shown to be markedly upregulated after microglial activation; thus, chemotaxis after injury could be mediated by this receptor (Ohsawa et al., 2007).

Phagocytosis is the terminal removal of cellular debris by phagocytes. In vertebrates, phagocytosis is performed mostly by macrophages and other specialized innate immune cells engulfing the cellular debris in phagosomes, membrane protrusions that fuse with lysosomes for terminal degradation. Although phagocytosis is activated primarily by the expression of “eat-me” signals on the surface of damaged or dead cells, injured neurons leak diffusible UDP signals that activate P2Y_6_-dependent phagocytic signaling in microglia (Koizumi et al., 2007). UDP, which also acts on G_*q*_-coupled P2Y6 receptors, induces the expression of chemokines (Kim et al., 2011). In contrast to the positive effect of P2Y6, the stimulation of P2X7 or P1 receptors attenuates microglial phagocytosis (Fang et al., 2009; Bulavina et al., 2012). Phagocytosis by microglia is also regulated by the ratio of P2/P1 activation. The interplay between P2 and P1 receptor activation is controlled by a cascade of extracellular enzymes that dephosphorylate purines, resulting in the formation of adenosine. In microglia, the capacity to degrade ATP and ADP depends on the expression of ecto-nucleoside triphosphate diphosphohydrolase 1 (E-NTPDase1, also called CD39). Deletion of CD39 practically abolishes ATP degradation and increases microglial phagocytic activity (Bulavina et al., 2012). Interestingly, the P2X7 receptor has also been detected in phagosomes. Lipids stimulate both actin assembly and the transport of ADP across the phagosomal membrane into the lumen. In the lumen, ADP is converted to ATP by adenylate kinase activity and activates P2X7 receptors in phagosomes, triggering actin assembly on the cytoplasmic membrane surface (Kuehnel et al., 2009a,b). These data indicate a distinct role of intracellular P2X7 receptors in phagocytosis.

In addition to its role in normal CNS function, ATP signaling is involved in neuroinflammation in a broad range of CNS pathologies (Di Virgilio et al., 2009). The extracellular concentration of ATP increases dramatically during inflammation (Idzko et al., 2007; Pellegatti et al., 2008), and P2X7 receptors are overexpressed in microglial cells in the neuroinflammatory foci of numerous neurodegenerative conditions (Weisman et al., 2012). The overexpression of P2X7 receptors in microglia, in the absence of a pathological insult, is sufficient to drive the activation and proliferation of microglia (Figure 1), which depends on the pore-forming capacity of this receptor (Monif et al., 2009). The activation of P2X7 receptors is coupled to the maturation and secretion of the key pro-inflammatory cytokine IL-1β [Ferrari et al., 1996; reviewed in Di Virgilio et al. (2009)], a signaling pathway that depends on P2X7 coupling with pannexin-1 and subsequent casapse-1 activation (Pelegrin and Surprenant, 2006). Activation of the large-pore P2X7 receptor also leads to the release of TNFα (Suzuki et al., 2004), the endocannabinoid 2-arachidonylglycerol (Witting et al., 2004), and superoxide (Parvathenani et al., 2003). However, whether the pore capacity of P2X7 depends on its coupling with pannexin-1 is a matter of debate. In neurons and astrocytes, pannexin1 appears to be the molecular substrate for the permeabilization pore (or death receptor channel) recruited into the P2X7 receptor signaling complex (Locovei et al., 2007; Silverman et al., 2009). In innate immune cells, including microglia and macrophages, P2X7 signaling appears to be independent of pannexin-1 (Hanley et al., 2012; Rigato et al., 2012). Thus, microglia proliferate and phagocytose dying motor neurons during early embryonic spinal cord invasion (Rigato et al., 2011). Notably, microglial invasion and proliferation are controlled by P2X7 receptor signaling in a pannexin-1-independent manner (Rigato et al., 2012). The pore dilation capacity of P2X7 receptors in microglia does not depend on the expression of pannexin-1 at this embryonic stage (Rigato et al., 2012).

The main function of adenosine receptors is the control of innate immune function. Adenosine receptors A_1_ and A_3_ block TNFα release by microglia, whereas A_2B_ stimulates the production and secretion of anti-inflammatory cytokine IL-10 (Koscsó et al., 2012), indicating an anti-inflammatory role of P1 receptor activation in the brain. Signaling through adenosine receptor A_2A_ drives the proliferation of spinal microglia after nerve injury (Bura et al., 2008), and intrathecal blockade of this receptor has been shown to abolish neuropathic pain in the same model (Loram et al., 2009). Finally, adenosine can suppress inflammation and aid in tissue restitution, in part, by promoting alternative macrophage activation. Alternative activation occurs in a Th2 cytokine environment and promotes the immunomodulatory and anti-inflammatory, rather than pro-inflammatory, properties of macrophages/microglia. Adenosine treatment of IL-4- or IL-13-activated macrophages augments the expression of alternative macrophage markers, primarily through the activation of A_2B_ receptors, though A_2A_ receptors also contribute to the effect (Csóka et al., 2012). Acting through A_3_ receptors, adenosine is also involved in the extension and migration of microglial processes (Ohsawa et al., 2012). Interestingly, simultaneous stimulation of P2Y12 and A_3_ receptors is required for microglial process extension, suggesting that intimate crosstalk occurs between P2Y12 and A_3_ receptors.

### Changes in ATP receptor expression in microglia

Microglia adopt an appropriate stimulus modality–dependent phenotype in response to injury or disease. The phenotypic catalog of microglia includes proliferative, migrational, and phagocytic responses, though how distinct the discrete molecular fingerprints of the phenotypes are is not clear (Hanisch and Kettenmann, 2007). Microglia undergo dramatic changes in shape and gene expression patterns within hours of *in vivo* activation, including modulation of the expression and function of purinergic receptors (Figure 1). Remodeling of purinoceptor expression has been observed *in situ* in various pathological models. Thus, epileptic seizures induced by kainate injections trigger an activation of microglia in hippocampal slices (Avignone et al., 2008), accompanied by an upregulation of the expression of mRNA specific for P2X1, P2X4, P2X7, P2Y6, P2Y12, and P2Y13 receptors. Functionally, this upregulation manifests as an increase in ATP-induced membrane currents and ATP-induced microglial motility (Avignone et al., 2008). Depending on the microglial stage, changes in purinergic expression determine the responses, sometimes with opposite effects, to extracellular purines. For example, the microglial chemotactic response to ATP is reversed following microglial activation. The switch from process attraction to repulsion is driven by upregulation of the G_s_-coupled A_2A_ receptor (Orr et al., 2009) concomitant with downregulation of the G_i_-coupled P2Y_12_ receptor (Haynes et al., 2006).

The extended and divergent time course of microglial activation suggests that the activation process is regulated by complex mechanisms, which may differ significantly depending on the initiating stimulus. *In vivo*, P2Y12 receptor expression decreases as microglia become activated after LPS injection in the striatum (Haynes et al., 2006). In contrast, facial-nerve axotomy, a classical model of microglial activation, induces an upregulation of microglial P2Y_12_ mRNA (Sasaki et al., 2003). Other stimuli, such as epileptic seizures or trauma, also lead to rapid upregulation of P2Y12 mRNA and protein in microglia (Franke et al., 2007; Avignone et al., 2008; Tozaki-Saitoh et al., 2008).

The P2X4R has been reported to be associated with the activation of microglia/macrophages after CNS injury and may play roles in inflammatory cascades involved in secondary brain damage. The development of mechanical allodynia temporally correlates with an increase in spinal P2X4R expression in microglia (Ulmann et al., 2008). Microglial P2X4R upregulation, the P2X4R^+^ state of microglia, seems to be common in most acute and chronic neurodegenerative diseases associated with inflammation. Microglial activation after traumatic brain injury also parallels a significant increase in P2X4R expression in microglia, which is suppressed by systemic treatment with dexamethasone (Zhang et al., 2007). The upregulation of microglial P2X4Rs has also been observed in animals expressing superoxide dismutase 1 mutant, an animal model of amyotrophic lateral sclerosis (D'Ambrosi et al., 2009), in the acute experimental autoimmune encephalomyelitis (EAE) model of multiple sclerosis (MS) (Guo and Schluesener, 2005), after spinal cord injury (Schwab et al., 2005), and in cerebral ischemia (Cavaliere et al., 2003). Different regulators of P2X4R expression in microglia have been described, such as the chemokine CCL2 (also known as monocyte chemoattractant protein, MCP-1; Biber et al., 2011; Toyomitsu et al., 2012), interferon-γ (Tsuda et al., 2009), and the extracellular matrix molecule fibronectin acting through Lyn kinase (Tsuda et al., 2008, 2009).

The role of P2X4R in microglial activation and how its expression affects microglial functions is unclear. In neurons, P2X4R influences inflammasome activation after spinal cord injury (de Rivero Vaccari et al., 2012). The inflammasome is a multiprotein complex that promotes the activation of caspase-1 and release of mature inflammatory cytokines, such as IL-1β and IL-18. This complex likely controls many aspects of neuroinflammatory processes. P2X4 knock-out mice exhibit impaired inflammasome signaling in the spinal cord, resulting in decreased IL-1β levels and reduced infiltration of neutrophils and monocyte-derived M1 macrophages, resulting in significant tissue sparing and improved functional outcomes (de Rivero Vaccari et al., 2012).

The metabotropic P2Y6R that controls microglial phagocytosis is highly expressed in surveying microglia, but it is also slightly upregulated in microglia following neuronal damage induced by kainic acid injection in the hippocampus (Koizumi et al., 2007), by trauma (Franke et al., 2007), or in animals expressing dismutase 1 mutant (D'Ambrosi et al., 2009). These data point to a role of this receptor in controlling the phagocytosis of necrotic cells after damage (Koizumi et al., 2007). In contrast, the phagocytosis of apoptotic cells prevents the spillover of cellular contents. Thus, whether UDP levels are sufficient to activate the phagocytosis of apoptotic cells or debris by microglia under normal physiological conditions remains to be determined.

Finally, the expression of adenosine receptors varies depending on microglial activation. Thus, surveying microglia express high levels of A_1_ and A_3_ receptors, and activation leads to their downregulation. In contrast, the expression level of the adenosine A_2A_ receptor in surveying/resting microglia is relatively low, but LPS dramatically increases its expression (Orr et al., 2009). The upregulation of A_2A_ receptor expression is also observed in pathological states, such as Parkinson disease and ischemia (Pedata et al., 2001; Schwarzschild et al., 2006).

## Glutamate receptors

### Expression of glutamate receptors in microglia

Glutamate is the major excitatory neurotransmitter of the CNS and perturbations in this transmitter's homeostasis have been reported in most neurodegenerative diseases. Glutamate activates both ionotropic and metabotropic receptors. Ionotropic receptors are classified into a-amino-3-hydroxy-5-methyl-4-isoxazolepropionic acid (AMPA), kainate, and *N*-methyl-d-aspartate (NMDA) subtypes according to their preferred agonist. Molecular cloning has revealed that each receptor subtype is composed of several subunits with high homology within each receptor class. Thus, AMPA receptors are formed by GluR1-4, kainate receptors by GluR5-7 and KA1-2, and NMDA receptors by NMDAR1, NMDAR2A-D, and NMDAR3A-B subunits (Cull-Candy and Leszkiewicz, 2004). AMPA receptors are activated by AMPA and kainate, whereas kainate receptors are activated by kainate and are best functionally isolated in the presence of GYKI53655, a selective AMPA receptor antagonist (Lerma, 2003). Similarly, metabotropic GluRs (mGluRs) can be classified as group I (mGluR1, mGluR5), group II (mGluR2, mGluR3), and group III (mGluR4, mGluR6-8) seven transmembrane receptors (Swanson et al., 2005). The individual mGluR groups are coupled to various G-proteins that activating phospholipase C (PLC, group I) or inhibit adenylate cyclase (groups II and III).

Few studies have characterized the functional expression of ionotropic glutamate receptors in microglial cells. An early study showed inward currents corresponding to the activation of low-Ca^2+^ permeability AMPA-type glutamate receptors (expressing the GluR2 subunit) in cultured microglia, the activation of which leads to TNF-α release [Noda et al., 2000; reviewed by Pocock and Kettenmann (2007)]. AMPA-type glutamate receptor activation also leads to *c-fos* expression (Eun et al., 2004). In contrast, no direct evidence has been found for the functional expression of kainate receptors in microglial cells. In contrast to *in vitro* conditions, electrophysiological recordings of microglial cells in retina or hippocampus slices clamped at −50 mV failed to detect any inward or outward current in response to glutamate or AMPA (Wu and Zhuo, 2008; Fontainhas et al., 2011). The expression of functional NMDA receptors in microglia in normal brain has not been reported, but the activation of microglia after the induction of transient forebrain ischemia leads to NMDAR1 subunit upregulation (Gottlieb and Matute, 1997). The functional significance of NMDA receptor upregulation in microglia is still unknown. However, NMDA injection into the somatosensory cortex of newborn rats triggers transient microglial activation (Acarin et al., 1996), whereas systemic administration of MK-801 prevents rapid microglial activation in the hippocampus, secondary to ischemic insults (Streit et al., 1992) or LPS treatment (Thomas and Kuhn, 2005). Whether NMDA receptor activation controls microglial activation directly or indirectly remains to be determined. Therefore, additional studies are necessary to characterize the existence of functional ionotropic glutamate receptors in the resident and activated microglia of slices, which could respond to glutamate release during synaptic activity or damage.

Regarding metabotropic glutamate receptors, different subunits of metabotropic groups I (mGluR5), II (mGluR2 and 3), and III (mGluR4, 6, and 8, but not mGluR7) are expressed by microglia and regulate microglial transformation into neuroprotective (via group III mGluRs) or neurotoxic (via group II mGluRs) phenotypes (Biber et al., 1999; Taylor et al., 2002, 2003, 2005; Pocock and Kettenmann, 2007). Microglial activation of group II mGluRs, particularly mGluR2, induces TNF-α and Fas ligand release, which trigger neuronal caspase-3 activation via TNFR1 (also known as p55) and Fas receptor, leading to neuronal death (Taylor et al., 2005). However, an agonist of mGluR3, a member of group II, has been shown to inhibit the toxicity of microglia toward oligodendrocytes (Pinteaux-Jones et al., 2008). Activation of groups II and III metabotropic glutamate receptors also modulates LPS-induced glutamate release by the xCT antiporter in microglia (McMullan et al., 2012), suggesting a neuroprotective role of its activation. Groups I and III metabotropic glutamate receptors also modulate the activity of NADPH oxidases, the main source of superoxide anions (Mead et al., 2012).

### Functions of glutamate receptors in microglia

Similar to ATP, glutamate is a chemotactic neurotransmitter for microglia. Microglia stimulated by kainate, via either AMPA or kainate receptors, undergo dramatic morphological and cytoskeletal changes characterized by the condensation of cytoplasmic actin filaments, rapid depolymerization and repolymerization, and cytoplasmic redistribution of condensed actin bundles (Christensen et al., 2006). Actin filament rearrangement is thought to be involved in locomotion and phagocytosis and to indicate an increased level of activation. Microglia cells exposed to glutamate exhibit increased cell membrane ruffling and migrate to a source of glutamate in cell culture and spinal cord slices. This chemotaxis is mediated by AMPA and metabotropic glutamate receptors on the microglia, and is dependent on the redistribution of actin filaments and tubulin following receptor activation (Liu et al., 2009).

However, the role of glutamate in regulating baseline motility remains controversial. Initial studies showed that neuronal neurotransmission and activity-dependent synaptic plasticity does not affect surveying microglial motility in the hippocampus (Wu and Zhuo, 2008). However, other studies have suggested that endogenous glutamatergic neurotransmission positively regulates the dendritic morphology and process motility of surveying microglia (Fontainhas et al., 2011). The processes of surveying microglia have been demonstrated to make brief (~5 min) and direct contacts with neuronal synapses at a frequency of approximately once per hour. These contacts are activity-dependent and reduce in frequency with reduced neuronal activity (Wake et al., 2009; Li et al., 2012). Neuronal activity affects the direction but not the basal level of microglial process motility (Li et al., 2012), which could explain the previous discrepancy with the article by Wu and Zhuo. Thus, neuronal activity steers surveying/resting microglia and facilitates their contacts with highly active neurons. This effect is not direct, as microglia do not express glutamate receptors in processes and lack direct responses to glutamatergic agonists *in situ*. Instead, these influences are mediated indirectly via extracellular ATP, which is released in response to glutamatergic neurotransmission through probenecid-sensitive pannexin hemichannels (Fontainhas et al., 2011; Li et al., 2012). The consequences of these microglia-synapse contacts depend probably on the nature and intensity of the stimulus. After transient cerebral ischemia, the duration of these microglia–synapse contacts is markedly prolonged (~1 h) and is frequently followed by the disappearance of the presynaptic bouton (Wake et al., 2009). However, an increase in spontaneous neuronal activity (i.e., by glutamate uncaging or kir channels overexpression in neurons) leads to the formation of microglial bulbous endings contacts with neurons that, surprisingly, reduce the activity of contacted neurons. This study suggests a role of surveying microglial in homeostatic regulation of neuronal activity (Li et al., 2012). These results suggest that microglia vigilantly monitor and respond to the functional status of synapses either to eliminate dysfunctional synapses or to silence them. Additional studies are needed to explore the mechanism and neurotransmitter involved in microglial detection of the functional status of synapses.

Glutamate is involved in the transmission of the death signal to microglia. Using the optically transparent larval zebrafish brain, rapidly propagating Ca^2+^ waves have been shown to determine the range of microglial responses to neuronal cell death. Though Ca^2+^-mediated microglial responses require ATP, the spreading of intercellular Ca^2+^ waves is ATP independent, and glutamate has been identified as a potent inducer of Ca^2+^-transmitted microglial attraction. Thus, the real-time analysis revealed the existence of a mechanism controlling microglial-targeted migration to neuronal injuries initiated by glutamate and proceeding across the brain in the form of a Ca^2+^ wave (Sieger et al., 2012).

## Role of microglial neurotransmitter receptors in pathology

Early during a pathological process, microglia may be stimulated by either non-self pathogens (stranger signals) or injured-self components (danger signals). Both stimuli can activate pattern-recognition receptors, such as the Toll-like receptors, scavenger receptors, and NOD system. The effector outputs of these stimuli focus on the clearance of tissue debris, generation of cues for tissue restoration, and resistance to pathogens. Together, these reactions comprise innate immune responses. Subsequent events may require the establishment of responses, including lymphocyte effector functions, antibodies, and immunological memory—collectively termed adaptive immunity. Microglial cells contribute to this process with antigen presentation, including the instruction of T cells to adopt varied effector programs (Th1, Th2, Th17) and, in some cases, directing them to the tissue from which the pathogenic material originated.

Although the innate immune response is beneficial in principal, an excessive and sustained activation of microglial cells is detrimental to neurons and oligodendrocytes (Merrill et al., 1993; Bezzi et al., 2001). Microglia activation has been described extensively in most pathological conditions in the CNS (Block and Hong, 2005), though its role is still debated (Schwartz et al., 2006). The outcome of microglial activation is complex and likely dependent on context. Microglia and macrophages can be activated by the cytokines interferon-g (IFN-g), IL-17, or LPS to a pro-inflammatory phenotype (M1), whereas IL-4 or IL-13 induce a state of alternative activation (M2) that is associated with neuroprotective functions that promote repair (Butovsky et al., 2006; Ponomarev et al., 2007; Kawanokuchi et al., 2008). Understanding the different processes and regulators of microglial activation will be important to unraveling their many complex functions. The thinking behind this dichotomization is that understanding these two microglial responses may minimize the harmful effects and capitalize on the beneficial effects (Popovich et al., 2011). In addition, a given facet of microglial activation that is beneficial in principle, such as phagocytosis, could turn detrimental under other circunstances. For example, phagocytosis under inflammatory conditions actively induces neuronal death (Neher et al., 2011) because inflammation causes viable neurons to express the “eat-me” signal, phosphatidylserine, on their surface, leading to their death through phagocytosis.

## Role of purinergic receptors in CNS injury

Purinergic signaling regulates both innate and adaptive immune responses and is involved in numerous acute insults and chronic neurodegenerative diseases of the CNS (Burnstock, 2008) because purine homeostasis is compromised in most diseases. However, relatively few studies have described the specific contribution of purinoceptor signaling pathways in microglia to neuropathology. In this section we summarize the data demonstrating the beneficial role of blocking P2 and P1 receptors, particularly P2X4R and P2X7R, in different CNS pathologies.

### P2 receptors in neuropathic pain: P2X4

Spinal microglia react and undergo a series of changes that directly influence the establishment of neuropathic pain states. Purinergic signaling via P2X4R is at the center of this reactivity (Beggs et al., 2012). Microglial P2X4 upregulation determines the behavioral manifestations of neuropathic pain arising from peripheral nerve injury and is sufficient to convert the response to normal non-painful peripheral inputs, from innocuous to nociceptive (review in Beggs et al., 2012). These findings are the basis for the concept that the microglial phenotype characterized by dramatic upregulation of P2X4Rs. The P2X4R^+^ state, is critical in the etiology of neuropathic pain (Beggs et al., 2012). However, other microglial purinergic receptors, such as P2X7R, P2Y12R, and P1 receptors, are also involved in neuropathic pain. The blockage of P2X7Rs has been shown to alleviate chronic pain in three different models of neuropathic pain (Honore et al., 2009). Microglial P2X7Rs might participate in the neuronal hyperexcitability of dorsal horn neurons and development of neuropathic pain through the production of pro-inflammatory cytokines and chemokines (Tsuda et al., 2013). P2X7R also participates in microglial P2X4R trafficking and assembly (Boumechache et al., 2009), which could indirectly modulate P2X4R-regulated neuropathic pain. In macrophages and microglia, P2X4 and P2X7 form homotrimers that interact. P2X7Rs are found predominantly at the cell surface, whereas P2X4Rs are primarily intracellular. However, microglial activation induces a rapid translocation of P2X4R to the surface, which is an efficient means of enhancing the function of P2X4R (Boumechache et al., 2009).

P2Y12R expression levels are also dramatically increased in microglial cells in the spinal cord after peripheral nerve injury, and the blockage of P2Y12Rs alleviates neuropathic pain (Kobayashi et al., 2008; Tozaki-Saitoh et al., 2008). After nerve injury, microglia are more abundant in layers II-III of the dorsal horn than in other areas, and some of them adhere to and engulf both injured and uninjured myelinated axons. This microglial engulfment is controlled by P2Y12R activation and directly involved in tactile allodynia (Maeda et al., 2010). The upregulation of other metabotropic P2 receptors, such as P2Y6R, P2Y13R, and P2Y14R, after nerve injury was reported recently (Kobayashi et al., 2012), and the concomitant block of the three receptors results in a longer suppressive effect on pain behavior (Kobayashi et al., 2012). In view of the crucial role played by different purinergic receptors in neuropathic pain, investigating the mechanisms of ATP release and how to modulate it as a means of attenuating neuropathic pain will be important.

Interestingly, a recent paper describes the involvement of microglial P2X4Rs in hyperalgesia produced by the gold-standard opiate analgesics. Morphine and other opiates are indispensable in the treatment of moderate-to-severe postoperative and chronic pain, but the use of these drugs is plagued by the development of two major problems: hyperalgesia and tolerance. Hyperalgesia is a sensitization process in which opioids, paradoxically, cause pain hypersensitivity. The spinal dorsal horn lamina I neurons are central targets for the analgesic effects of morphine and other opiates, and mediate morphine-induced hyperalgesia and tolerance. In particular, morphine induces analgesia via inhibition in lamina I neurons. In contrast, morphine induces hyperalgesia via the P2X4R-BDNF-KCC2 disinhibition cascade between microglia and lamina I neurons. Thus, BDNF release by activation of P2X4Rs in microglia impairs Cl^−^ homeostasis by downregulating K^+^–Cl^−^ co-transporter KCC2 in those neurons (Ferrini et al., 2013).

P2X4Rs are also involved in other acute insults. Thus, the activation of microglia after hypoxia in the neonatal rat brain, a model of periventricular white matter damage, is mediated by P2X4R signaling (Li et al., 2011). P2X4 is also upregulated in microglial cells in the CA1 and transition zone to CA2 regions of the hippocampus after ischemia (Cavaliere et al., 2003), and its blockade confers neuronal protection (Cavaliere et al., 2005). Conversely, activation of P2X4 purinergic signaling in glia after traumatic injury stimulates the synthesis and release of thrombospondin-1, an extracellular matrix molecule that induces synapse formation during development, and it may play a role in CNS repair and remodeling after injury (Tran and Neary, 2006).

### P2X7 receptors: a promising target for neuroprotection

In immune cells, P2X7R activation promotes assembly of the inflammasome, and caspase-1-dependent cleavage and release of biologically active IL-1β and IL-18 *in vitro* and *in vivo*, ultimately leading to a rapid form of cell death called pyroptosis (Miao et al., 2011). P2X7R antagonists improve neuronal viability by inhibiting P2X7R-activated NLRP3 inflammasome formation and the subsequent IL-1β release from glia (Murphy et al., 2012). P2X7R stimulation in neurons also induces inflammasome activation in these cells (Silverman et al., 2009). In addition, P2X7R activation in neurons and oligodendrocytes leads to a massive calcium influx that induces mitochondrial damage and initiates the apoptotic cascade (Matute et al., 2007; Díaz-Hernández et al., 2009; Arbeloa et al., 2012). P2X7Rs have unique properties that could be relevant to pathological conditions. First, P2X7Rs have high Ca^2+^ permeability, similar to that of NMDA receptors. Second, in contrast to NMDA receptors, P2X7Rs can be activated at resting membrane potentials and do not require membrane depolarization. Finally, P2X7Rs do not desensitize and open a large pore that causes cytolytic cell death after prolonged activation (Surprenant et al., 1996). Prolonged activation of P2X7Rs kills all CNS cells, including neurons (Jun et al., 2007; Díaz-Hernández et al., 2009; Arbeloa et al., 2012), oligodendrocytes (Matute et al., 2007), astrocytes (Kim et al., 2011), and microglia (Harada et al., 2011).

Acute insults, such as trauma and ischemia, lead to a massive release of nucleotides from disrupted cells at a level sufficient to activate low-affinity P2X7Rs in neighboring neurons and oligodendrocytes, leading to their death by excitotoxicity (Wang et al., 2004; Domercq et al., 2010; Arbeloa et al., 2012). Both insults induced the activation of microglia and dramatic remodeling of purinoceptors in microglia, which could influence microglial functions with beneficial or detrimental consequences. P2X7R antagonists modulate microglial activation and the inflammatory response after ischemia, which could contribute to the therapeutic value of these antagonists (Melani et al., 2006; Chu et al., 2012). Cerebral microvascular occlusion elicits microvascular injury, mimicking the different degrees of stroke severity observed in patients. Recently, a role of microglial P2X7R in this type of injury has been proposed. After inducing focal microsphere embolism to microvessels, microglia are recruited to the lesion site through a P2X7R-dependent mechanism and release FasL contributing to neuroinflammation (Lu et al., 2012). The microglial response to P2X7R activation appears to be region specific. Thus, in the status epilepticus (SE), microglia appear amoeboid or phagocytic in the dentate gyrus (DG) and piriform cortex due to P2X7R activation, but elongated in the CA1 hippocampal regions and frontoparietal cortex (Choi et al., 2012).

P2X7R antagonists have also been protective in animal models of MS, amyotrophic lateral sclerosis, Parkinson's disease, Huntington's disease, and Alzheimer's disease (Table 2). Whether protection is mediated by blocking neuronal or oligodendroglial excitotoxicity, inflammation, or both remains to be determined in most neurodegenerative diseases. In Alzheimer's disease, different mechanisms determine the beneficial effects of P2X7R antagonists. *In vivo* administration of P2X7R antagonists reduce amyloid plaque formation through a signaling cascade involving the activation of GSK-3 kinase and increased non-amyloidogenic amyloid precursor protein processing by α-secretase (Diaz-Hernandez et al., 2012). However, P2X7Rs are necessary for β-amyloid-induced microglial activation (Sanz et al., 2009). The treatment of chronic EAE, the animal model of MS, with P2X7R antagonists reduces demyelination and ameliorates associated neurological symptoms (Matute et al., 2007). Because ATP signaling can trigger oligodendrocyte excitotoxicity (Matute et al., 2007), the beneficial effect of P2X7R antagonists in this pathology may be due its protective role in oligodendrocytes and axons, more than the possible interference of the immune system. Importantly, sustained activation of P2X7Rs *in vivo* causes lesions that are reminiscent of the major features of MS plaques, and P2X7 RNA and protein levels are elevated in normal-appearing axon tracts in MS patients, suggesting that oligodendroglial signaling through P2X7Rs is enhanced in MS, which may render this cell type more vulnerable to ATP dysregulation (Matute et al., 2007).

**Table 2:** **Neuroprotective properties of P2X7 receptor antagonists**.

**Disease**	**P2X7 receptor involvement**	**References**
**CHRONIC NEURODEGENERATION**		
Alzheimer's disease	P2X7 mediates microglial neuroinflammatory reaction in different models of Alzheimer's disease	Parvathenani et al., 2003; Rampe et al., 2004; McLarnon et al., 2006
	P2X7 receptor blocks α-secretase activity/P2X7 triggers α-secretase activity	Delarasse et al., 2011; León-Otegui et al., 2011
	*In vivo* P2X7 inhibition reduces amyloid plaques in Alzheimer's disease	Diaz-Hernandez et al., 2012
	Upregulation of P2X7 in microglia in the cerebral cortex of the APPswe/PS1dE9 mice, a mouse model of AD	Lee et al., 2011
Amyotrophic lateral sclerosis	P2X7 receptor activation in spinal cord SOD1(G93A) astrocytes leads to motor neuron death	Gandelman et al., 2010
	P2X7 is overexpressed in activated microglial in ALS	Yiangou et al., 2006
Parkinson's disease	ATP mediates necrotic cell death in SN4741 dopaminergic neurons though P2X7 receptors	Jun et al., 2007
	P2X7 increases in astrocytes in the rotenone Parkinson's disease model	Gao et al., 2011
Huntington's disease	P2X7 antagonists prevented neuronal apoptosis in HD mice	Díaz-Hernández et al., 2009
Multiple sclerosis	P2X7^−/−^ mice are more susceptble to EAE, the MS model	Chen and Brosnan, 2006; Witting et al., 2006
	P2X7 mediates ATP excitotoxicity to oligodendrocytes and P2X7 blockage improves neurological damage in EAE	Matute et al., 2007
	Association of gain of function P2X7 variants with MS	Oyanguren-Desez et al., 2011
**ACUTE INSULTS**		
Epilepsy	Enhanced purinergic signaling in microglia in status epilepticus	Avignone et al., 2008
	P2X7^−/−^mice and Panx1 gene silencing showed greater susceptibility to pilocarpine-induced seizures	Kim and Kang, 2011
	P2X7 antagonists as well Panx1 gene silencing blocked status epilepticus induced by kainic acid	Santiago et al., 2011; Engel et al., 2012
	P2X7 antagonists prevented astroglial apoptosis in status epilepticus	Kim et al., 2009
Ischemia	P2X7 receptors is overexpressed in activated microglia and in neurons in different models of *in vitro* and *in vivo* ischemia	Cavaliere et al., 2004, 2005; Franke et al., 2004
	P2X7 antagonists reduces neuronal damage and infarct size after transient focal ischemia	Le Feuvre et al., 2003; Melani et al., 2006; Arbeloa et al., 2012
	P2X7 blockage amielorates oligodendroglial and axonal damage after white matter ischemia	Domercq et al., 2010
Trauma	P2X7 receptor inhibition improves recovery after spinal cord injury	Wang et al., 2004; Peng et al., 2009

P2X7Rs have a low affinity for ATP (100 μM-10 mM), and ATP levels in the extracellular space are in the low nanomolar range due to its rapid inactivation by powerful ubiquitous ecto-ATPases, whether this receptor is activated under physiological conditions is unclear. However, ATP is available at high concentrations within the cytoplasm (1–3 mM) and quickly released in sufficient quantities to activate P2X7Rs following cell damage in acute insults and chronic neurodegenerative diseases. These characteristics indicate an almost exclusive activation of P2X7Rs in pathological states and a low or negligible interference with normal brain functionality. Therefore, P2X7R could be an ideal therapeutic target for neurodegenerative diseases.

### Adenosine receptors

Adenosine plays a relevant role as a neuromodulator and, thus, contributes to MS, a chronic disease with an autoimmune and inflammatory basis. A_1_ receptor-null mice have been shown to develop more severe demyelination and motor symptoms in chronic EAE compared to their wild-type counterparts (Tsutsui et al., 2004). The aggravation of EAE is mostly mediated by cells in the microglial lineage and involves the release of toxic factors by macrophages/microglia lacking A_1_ receptors (Tsutsui et al., 2004). More recently, a protective role of adenosine A_2A_ receptors was reported in this model. Genetic inactivation of the A_2A_ receptor exacerbates EAE pathology in mice. In addition, A_2A_ receptor knockout mice display increased inflammatory cell infiltration and enhanced microglial cell activation in the cortex, brainstem, and spinal cord (Mills et al., 2012; Yao et al., 2012).

Inflammation also contributes to post-ischemic delayed cerebral damage. A_3_ adenosine receptor expression is modulated by the activation state of inflammatory cells and, in turn, its activation regulates the inflammatory activity of immune cells (Bar-Yehuda et al., 2007; Ochaion et al., 2009). The administration of A_3_ agonists before or immediately after ischemic insults has been shown to significantly protect the brain in rodent ischemia models (Von Lubitz et al., 1994, 2001; Chen et al., 2006). Importantly, A_3_ agonists protect against ischemic brain injury when applied 7 h after the ischemic insult (5.5 h after starting reperfusion) (Choi et al., 2011). The effect could be due to an inhibitory effect of the A_3_ agonist on microglial/monocyte migration through the regulation of Rho GTPases (Choi et al., 2011).

## Role of glutamate receptors in CNS injury

The possible contribution of microglia glutamate signaling to pathology has not been analyzed thoroughly. Few studies have demonstrated that the activation of ionotropic glutamate receptors in microglia has deleterious consequences to neurons and oligodendrocytes. NMDA receptor expression is upregulated in activated microglia following ischemia (Gottlieb and Matute, 1997; Kaur et al., 2006), which contributes to oligodendrocyte damage in hypoxic postnatal rats, a model of periventricular white matter damage. The activation of NMDA receptors in microglia leads to NO release in response to NF-kb signaling, which is known to induce oligodendrocyte cell death (Li et al., 2005). Pharmacological inhibition of NMDA receptors (MK801), NF-kb (BAY), and iNOS (1400w) prevents oligodendrocyte cell death (Murugan et al., 2011). Thus, NMDA receptor blockade protects oligodendrocytes by reducing the release of NO from microglia (Tahraoui et al., 2001; Murugan et al., 2011), in addition to the effect of direct blockage of NMDA receptors in these cells (Manning et al., 2008). In contrast, kainate-activated microglia induce IL-1β and TNF-α release, which mediate increased excitability of hippocampal CA3 neurons (Zheng et al., 2010; Zhu et al., 2010), an effect that could be relevant in acute insults.

The activation of microglial metabotropic receptors has been reported to regulate superoxide production by modulating NADPH oxidase (Nox) activity. Nox enzymes are major generators of ROS, which contribute to the progression of CNS disorders as diverse as amyotrophic lateral sclerosis, schizophrenia, Alzheimer's disease, Parkinson's disease, and stroke. Microglia are the predominant cells expressing Nox enzymes (Harrigan et al., 2008). Nox activation is elicited by agonists of metabotropic mGlu3 receptors, promotes neurotoxicity, and is inhibited by antagonists of mGluR5 receptors (Mead et al., 2012). For example, the regulation of Nox activity by mGluRs could contribute to limiting microglial activation after traumatic brain injury, improving motor and cognitive recovery (Byrnes et al., 2012).

## Role of microglia in glutamate and ATP homeostasis and its contribution to neurodegenerative diseases

### ATP homeostasis

ATP homeostasis is compromised in most CNS pathologies. Immediately after acute CNS injury, astrocytes and damaged cells release ATP, resulting in rapid activation of microglia. ATP and UTP are released by apoptotic cells as a “find-me” signal in the earliest stages of death to recruit phagocytes to the plasma membrane channel pannexin 1 (PANX1). Pharmacological inhibition and siRNA-mediated knockdown of PANX1 leads to decreased nucleotide release and monocyte recruitment by apoptotic cells (Chekeni et al., 2010). Pannexins also open following ischemic insult (Thompson et al., 2006; Domercq et al., 2010; MacVicar and Thompson, 2010), in response to high extracellular K^+^ (Silverman et al., 2009), after NMDA receptor stimulation (Thompson et al., 2008), and, surprisingly, in response to caspase cleavage (Chekeni et al., 2010), which suggests that pannexins may open in most pathological contexts. However, the expression of pannexins in microglia and their possible influence on ATP release under normal conditions and after microglial activation has not been characterized. Finally, microglia are able to release ATP after activation with LPS, leading to an increase in excitatory neurotransmission (Pascual et al., 2012). Different mechanisms have been proposed, including microglial release of ATP via zinc uptake by zinc transporters (Higashi et al., 2011). In addition, lysosomes in microglia contain abundant ATP and exhibit Ca^2+^-dependent exocytosis in response to various stimuli (Dou et al., 2012).

### Glutamate homeostasis

Since the discovery of excitotoxicity and its contribution to neuronal cell death in neuropathology, numerous studies have aimed to understand the origin of alterations in glutamate homeostasis, which determines lethal overactivation of glutamate receptors. *In vitro* studies have demonstrated that activated microglia play a deleterious role by releasing glutamate or altering its homeostasis. Surprisingly, a recent study demonstrated a neuroprotective role of surveying microglia in excitotoxicity-induced neurodegeneration in the hippocampus (Vinet et al., 2012). This region exhibits differential sensitivity to excitotoxicity, with the CA1 region more vulnerable than CA3 or DG neurons. However, ablation of ramified microglia in the latter two areas exacerbates neuronal cell death, suggesting a protective role of surveying microglia in these areas (Vinet et al., 2012). The mechanism by which surveying microglia confer protection remains to be determined.

Microglia, mainly in the activated state, contribute to alterations in neurotransmitter homeostasis via three main mechanisms: (1) release of excitotoxins, including glutamate (Piani et al., 1992), quinolinate (Heyes et al., 1996), D-serine (Wu et al., 2004), and ATP; (2) interfering with glutamate uptake, which is mainly carried out by astrocytes, leading to extracellular glutamate accumulation; or (3) altering astrocyte gliotransmitter release (including glutamate) or synaptic transmission.

A key determinant of microglial neurotoxicity is the release of excitotoxins, such as glutamate. The vast majority of glutamate exported from activated microglia can be attributed to the x^−^_c_ exchange mechanism (a.k.a., SLC7A11 or CCBR1). This antiporter is a membrane-bound, Cl^−^-dependent, Na^+^-independent antiporter that mediates the cellular uptake of cystine in exchange for glutamate at a 1:1 ratio, primarily following the relative concentration gradients of each of these amino acids (Bridges et al., 2012). This mechanism becomes extremely active in microglia because it is the primary route for internalizing cystine, which is converted to cysteine intracellularly, the rate-limiting substrate in glutathione synthesis (Bridges et al., 2012). As activated microglia produce ROS, they place themselves under severe oxidative stress. Although the bulk of superoxide produced by Nox is released from microglia, some remains intracellular. Thus, the microglial oxidative burst creates a GSH shortage that is alleviated by cystine influx through the x^−^_c_ antiporter, extruding glutamate in the balance (Barger et al., 2007). The obligate exchange of glutamate could be deleterious to neuronal cells and other tissues that are susceptible to excitotoxic damage. Accordingly, the cystine/glutamate exchanger has been implicated in glutamate-associated disorders, such as glioma-derived epileptic seizures (Buckingham et al., 2011), oxidative glutamate toxicity (Oka et al., 1993; Albrecht et al., 2010), Alzheimer's disease (Barger and Basile, 2001; Qin et al., 2006), bacterial infection/LPS (Taguchi et al., 2007), MS (Domercq et al., 2007; Pampliega et al., 2011), Parkinson's disease, AIDS (Zeng et al., 2010), virally-induced encephalopathy (Espey et al., 1998; Qin et al., 2010), tumor proliferation (Ogunrinu and Sontheimer, 2010), antigen presentation (D'Angelo et al., 2010), and hypoxia (Fogal et al., 2007; Jackman et al., 2010).

**Figure 2:**
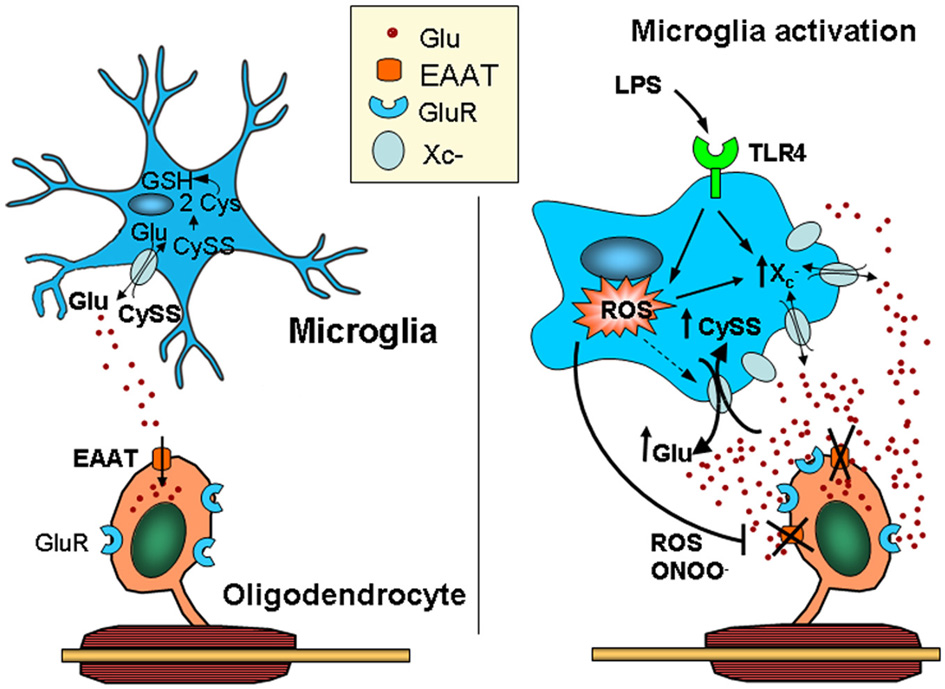
**Activated microglia can kill oligodendrocytes via a dual mechanism leading to glutamate excitotoxicity.** Microglia release glutamate, primarily through the cystine/glutamate exchange system (system x^−^_c_), which is highly active in these cells due to its high rate of reactive oxygen species (ROS) production. Thus, cystine is intracellularly converted into cysteine, the rate-limiting substrate in glutathione synthesis. Under physiological conditions, glutamate released by the exchanger is efficiently taken up by glutamate transporters expressed in surrounding cells, including astrocytes and oligodendrocytes. In contrast, microglia activated by pro-inflammatory stimuli (e.g., LPS acting at TLR4) release ROS and pro-inflammatory cytokines that impair the function of glutamate transporters, such as TNF-α and IL-1β, elevating extracellular glutamate levels. In addition, the over-expression of system x^−^_c_ in activated microglia increases ambient glutamate concentrations. Together, these deleterious effects on glutamate homeostasis can result in excitotoxicity (Domercq et al., 2007). CySS, cystine; Glu, glutamate; LPS, lipopolysaccharide; TLR4, Toll-like receptor.

Both surveying and activated microglia release glutamate through the cystine/glutamate antiporter (Domercq et al., 2007) (Figure 2). However, glutamate released through surveying microglia is rapidly and efficiently removed by glutamate transporters in other glial cells, mainly astrocytes, though also oligodendrocytes (Domercq et al., 1999). In contrast, the activation of microglia induces the release of factors, such as ROS, TNF-α, and IL-1β, that impair the function of EAATs, resulting in an increase in the extracellular levels of glutamate (Domercq et al., 2007). In addition, autoantigen-activated myelin basic protein-specific T cells also inhibit EAATs (Korn et al., 2005), suggesting that these mechanisms could contribute to alterations in glutamate homeostasis in the plasma and cerebrospinal fluid of MS patients. Gliomas also achieve excitotoxic levels of glutamate through high levels of system x^−^_c_ activity coupled with a relative absence of sodium-dependent transport (Ye and Sontheimer, 1999; Ye et al., 1999; Kim et al., 2001; Takano et al., 2001; Rothstein, 2002; Chung et al., 2005). Glutamate released by system x^−^_c_ in gliomas triggers excitotoxic cell death in the regions surrounding the tumor, allowing the tumor cells to migrate and invade (Lyons et al., 2007). Interestingly, glutamate export by the microglial cystine/glutamate antiporter is inhibited by mGluRII and III metabotropic glutamate receptor activation (McMullan et al., 2012).

Finally, microglia may regulate astrocyte-dependent synaptic modulation, called gliotransmission (Perea et al., 2009; Pascual et al., 2012). The first evidence of gliotransmission came from the seminal observation that glutamate release from cultured astrocytes (in this case stimulated via CXCR4 chemokine receptors) is dramatically amplified by the presence of activated microglia in the astrocytic microenvironment (Bezzi et al., 2001). Constitutive TNFα was reported to control astrocyte gliotransmission under physiological conditions (Stellwagen and Malenka, 2006; Santello et al., 2011). However, microglia activated by LPS release massive concentrations of TNF-α (approximately 10-fold); at these high concentrations, TNF-α changes its mode of action, not only gating, but also directly causing glutamate release from astrocytes. This alternative TNF-α action is mediated by prostaglandin E_2_ (PGE_2_) and was found to induce slow excitotoxic neuronal damage, both in cell culture and *in vivo* (Bezzi et al., 2001).

## Conclusions

Neurotransmitter signaling is relevant to microglial functions. Microglia express receptors for neurotransmitters and contribute to neurotransmitter homeostasis. In particular, microglia are endowed with virtually all types of purinergic receptors that are differentially expressed at different stages and mediate process extension and retraction, cytokine release, migration, proliferation, and phagocytosis. In turn, activated microglia can release ATP and modulate synaptic activity. In addition, microglia also have an ample variety of glutamate receptors that mediate chemotaxis, ATP release, and surveillance of the functional status of synapses. Notably, microglia are critical controllers of glutamate homeostasis via the cystine/glutamate Xc- exchanger. Both ATP and glutamate receptors in microglia are relevant to neuroinflammation in various pathological conditions, including neurodegenerative diseases, and can be valuable targets for drug development in neuropathic pain and neuroprotection.

### Conflict of interest statement

The authors declare that the research was conducted in the absence of any commercial or financial relationships that could be construed as a potential conflict of interest.

## References

[B1] Abbracchio M. P. Ceruti S. (2007). P1 receptors and cytokine secretion. Purinergic Signal 3, 13–25 10.1007/s11302-006-9033-z 18404415 PMC2096764

[B2] Acarin L. González B. Castellano B. Castro A. J. (1996). Microglial response to N-methyl-D-aspartate-mediated excitotoxicity in the immature rat brain. J. Comp. Neurol. 367, 361–374 10.1002/(SICI)1096-9861(19960408)367:3<361::AID-CNE4>3.0.CO;2-3 8698898

[B3] Albrecht P. Lewerenz J. Dittmer S. Noack R. Maher P. Methner A. (2010). Mechanisms of oxidative glutamate toxicity: the glutamate/cystine antiporter system xc- as a neuroprotective drug target. CNS Neurol. Disord. Drug Targets 9, 373–382 10.2174/187152710791292567 20053169

[B4] Arbeloa J. Pérez-Samartín A. Gottlieb M. Matute C. (2012). P2X7 receptor blockade prevents ATP excitotoxicity in neurons and reduces brain damage after ischemia. Neurobiol. Dis. 45, 954–961 10.1016/j.nbd.2011.12.014 22186422

[B5] Avignone E. Ulmann L. Levavasseur F. Rassendren F. Audinat E. (2008). Status epilepticus induces a particular microglial activation state characterized by enhanced purinergic signaling. J. Neurosci. 28, 9133–9144 10.1523/JNEUROSCI.1820-08.2008 18784294 PMC6670931

[B6] Ballerini P. Di Iorio P. Ciccarelli R. Caciagli F. Poli A. Beraudi A. (2005). P2Y1 and cysteinyl leukotriene receptors mediate purine and cysteinyl leukotriene co-release in primary cultures of rat microglia. Int. J. Immunopathol. Pharmacol. 18, 255–268 15888248 10.1177/039463200501800208

[B7] Barger S. W. Basile A. S. (2001). Activation of microglia by secreted amyloid precursor protein evokes release of glutamate by cystine exchange and attenuates synaptic function. J. Neurochem. 76, 846–854 10.1046/j.1471-4159.2001.00075.x 11158256

[B8] Barger S. W. Goodwin M. E. Porter M. M. Beggs M. L. (2007). Glutamate release from activated microglia requires the oxidative burst and lipid peroxidation. J. Neurochem. 101, 1205–1213 10.1111/j.1471-4159.2007.04487.x 17403030 PMC1949347

[B9] Bar-Yehuda S. Silverman M. H. Kerns W. D. Ochaion A. Cohen S. Fishman P. (2007). The anti-inflammatory effect of A3 adenosine receptor agonists: a novel targeted therapy for rheumatoid arthritis. Expert Opin. Investig. Drugs 16, 1601–1613 10.1517/13543784.16.10.1601 17922624

[B10] Beggs S. Trang T. Salter M. W. (2012). P2X4R+ microglia drive neuropathic pain. Nat. Neurosci. 15, 1068–1073 10.1038/nn.3155 22837036 PMC5023423

[B11] Bezzi P. Domercq M. Brambilla L. Galli R. Schols D. De Clercq E. (2001). CXCR4-activated astrocyte glutamate release via TNFalpha: amplification by microglia triggers neurotoxicity. Nat. Neurosci. 4, 702–710 10.1038/89490 11426226

[B12] Biber K. Laurie D. J. Berthele A. Sommer B. Tölle T. R. Gebicke-Härter P. J. (1999). Expression and signaling of group I metabotropic glutamate receptors in astrocytes and microglia. J. Neurochem. 72, 1671–1680 10.1046/j.1471-4159.1999.721671.x 10098876

[B13] Biber K. Tsuda M. Tozaki-Saitoh H. Tsukamoto K. Toyomitsu E. Masuda T. (2011). Neuronal CCL21 up-regulates microglia P2X4 expression and initiates neuropathic pain development. EMBO J. 30, 1864–1873 10.1038/emboj.2011.89 21441897 PMC3101996

[B14] Block M. L. Hong J. S. (2005). Microglia and inflammation-mediated neurodegeneration: multiple triggers with a common mechanism. Prog. Neurobiol. 76, 77–98 10.1016/j.pneurobio.2005.06.004 16081203

[B15] Boucsein C. Zacharias R. Färber K. Pavlovic S. Hanisch U. K. Kettenmann H. (2003). Purinergic receptors on microglial cells: functional expression in acute brain slices and modulation of microglial activation *in vitro*. Eur. J. Neurosci. 17, 2267–2276 10.1046/j.1460-9568.2003.02663.x 12814360

[B16] Boumechache M. Masin M. Edwardson J. M. Górecki D. C. Murrell-Lagnado R. (2009). Analysis of assembly and trafficking of native P2X4 and P2X7 receptor complexes in rodent immune cells. J. Biol. Chem. 284, 13446–13454 10.1074/jbc.M901255200 19304656 PMC2679444

[B17] Brandenburg L. O. Jansen S. Wruck C. J. Lucius R. Pufe T. (2010). Antimicrobial peptide rCRAMP induced glial cell activation through P2Y receptor signalling pathways. Mol. Immunol. 47, 1905–1913 10.1016/j.molimm.2010.03.012 20392497

[B18] Braun N. Sévigny J. Robson S. C. Enjyoji K. Guckelberger O. Hammer K. (2000). Assignment of ecto-nucleoside triphosphate diphosphohydrolase-1/cd39 expression to microglia and vasculature of the brain. Eur. J. Neurosci. 12, 4357–4366 10.1111/j.1460-9568.2000.01342.x 11122346

[B19] Bridges R. Lutgen V. Lobner D. Baker D. A. (2012). Thinking outside the cleft to understand synaptic activity: contribution of the cystine-glutamate antiporter (system xc) to normal and pathological glutamatergic signaling. Pharmacol. Rev. 64, 780–802 10.1124/pr.110.003889 22759795 PMC3400835

[B20] Buckingham S. C. Campbell S. L. Haas B. R. Montana V. Robel S. Ogunrinu T. (2011). Glutamate release by primary brain tumors induces epileptic activity. Nat. Med. 17, 1269–1274 10.1038/nm.2453 21909104 PMC3192231

[B21] Bulavina L. Szulzewsky F. Rocha A. Krabbe G. Robson S. C. Matyash V. (2012). NTPDase1 activity attenuates microglial phagocytosis. Purinergic Signal. [Epub ahead of print]. 10.1007/s11302-012-9339-y 23208703 PMC3646122

[B22] Bura S. A. Nadal X. Ledent C. Maldonado R. Valverde O. (2008). A 2A adenosine receptor regulates glia proliferation and pain after peripheral nerve injury. Pain 140, 95–103 10.1016/j.pain.2008.07.012 18768260

[B23] Burnstock G. (2008). Purinergic signalling and disorders of the central nervous system. Nat. Rev. Drug Discov. 7, 575–590 10.1038/nrd2605 18591979

[B24] Butovsky O. Ziv Y. Schwartz A. Landa G. Talpalar A. E. Pluchino S. (2006). Microglia activated by IL-4 or IFN-gamma differentially induce neurogenesis and oligodendrogenesis from adult stem/progenitor cells. Mol. Cell. Neurosci. 31, 149–160 10.1016/j.mcn.2005.10.006 16297637

[B25] Byrnes K. R. Loane D. J. Stoica B. A. Zhang J. Faden A. I. (2012). Delayed mGluR5 activation limits neuroinflammation and neurodegeneration after traumatic brain injury. J. Neuroinflammation 28, 9–43 10.1186/1742-2094-9-43 22373400 PMC3308916

[B26] Cavaliere F. Amadio S. Sancesario G. Bernardi G. Volonté C. (2004). Synaptic P2X7 and oxygen/glucose deprivation in organotypic hippocampal cultures. J. Cereb. Blood Flow Metab. 24, 392–398 10.1097/00004647-200404000-00004 15087708

[B27] Cavaliere F. Dinkel K. Reymann K. (2005). Microglia response and P2 receptor participation in oxygen/glucose deprivation-induced cortical damage. Neuroscience 136, 615–623 10.1016/j.neuroscience.2005.04.038 16344139

[B28] Cavaliere F. Florenzano F. Amadio S. Fusco F. R. Viscomi M. T. D'Ambrosi N. (2003). Up-regulation of P2X2, P2X4 receptor and ischemic cell death: prevention by P2 antagonists. Neuroscience 120, 85–98 10.1016/S0306-4522(03)00228-8 12849743

[B29] Chekeni F. B. Elliott M. R. Sandilos J. K. Walk S. F. Kinchen J. M. Lazarowski E. R. (2010). Pannexin 1 channels mediate ‘find-me’ signal release and membrane permeability during apoptosis. Nature 467, 863–867 10.1038/nature09413 20944749 PMC3006164

[B30] Chen G. J. Harvey B. K. Shen H. Chou J. Victor A. Wang Y. (2006). Activation of adenosine A3 receptors reduces ischemic brain injury in rodents. J. Neurosci. Res. 84, 1848–1855 10.1002/jnr.21071 17016854

[B31] Chen L. Brosnan C. F. (2006). Exacerbation of experimental autoimmune encephalomyelitis in P2X7R-/- mice: evidence for loss of apoptotic activity in lymphocytes. J. Immunol. 176, 3115–3126 16493071 10.4049/jimmunol.176.5.3115

[B32] Choi H. K. Ryu H. J. Kim J. E. Jo S. M. Choi H. C. Song H. K. (2012). The roles of P2X7 receptor in regional-specific microglial responses in the rat brain following status epilepticus. Neurol. Sci. 33, 515–525 10.1007/s10072-011-0740-z 21845474

[B33] Choi I. Y. Lee J. C. Ju C. Hwang S. Cho G. S. Lee H. W. (2011). A3 adenosine receptor agonist reduces brain ischemic injury and inhibits inflammatory cell migration in rats. Am. J. Pathol. 179, 2042–2052 10.1016/j.ajpath.2011.07.006 21854743 PMC3181366

[B34] Christensen R. N. Ha B. K. Sun F. Bresnahan J. C. Beattie M. S. (2006). Kainate induces rapid redistribution of the actin cytoskeleton in ameboid microglia. J. Neurosci. Res. 84, 170–181 10.1002/jnr.20865 16625662

[B35] Chu K. Yin B. Wang J. Peng G. Liang H. Xu Z. (2012). Inhibition of P2X7 receptor ameliorates transient global cerebral ischemia/reperfusion injury via modulating inflammatory responses in the rat hippocampus. J. Neuroinflammation 9:69 10.1186/1742-2094-9-69 22513224 PMC3418181

[B36] Chung W. J. Lyons S. A. Nelson G. M. Hamza H. Gladson C. L. Gillespie G. Y. (2005). Inhibition of cystine uptake disrupts the growth of primary brain tumors. J. Neurosci. 25, 7101–7110 10.1523/JNEUROSCI.5258-04.2005 16079392 PMC2681064

[B37] Csóka B. Selmeczy Z. Koscsó B. Németh Z. H. Pacher P. Murray P. J. (2012). Adenosine promotes alternative macrophage activation via A2A and A2B receptors. FASEB J. 26, 376–386 10.1096/fj.11-190934 21926236 PMC3250237

[B38] Cull-Candy S. G. Leszkiewicz D. N. (2004). Role of distinct NMDA receptor subtypes at central synapses. Sci. STKE 2004:re16 10.1126/stke.2552004re16 15494561

[B39] D'Ambrosi N. Finocchi P. Apolloni S. Cozzolino M. Ferri A. Padovano V. (2009). The proinflammatory action of microglial P2 receptors is enhanced in SOD1 models for amyotrophic lateral sclerosis. J. Immunol. 183, 4648–4656 10.4049/jimmunol.0901212 19734218

[B40] D'Angelo J. A. Dehlink E. Platzer B. Dwyer P. Circu M. L. Garay J. (2010). The cystine/glutamate antiporter regulates dendritic cell differentiation and antigen presentation. J. Immunol. 185, 3217–3226 10.4049/jimmunol.1001199 20733204 PMC3100200

[B41] Davalos D. Grutzendler J. Yang G. Kim J. V. Zuo Y. Jung S. (2005). ATP mediates rapid microglial response to local brain injury *in vivo*. Nat. Neurosci. 8, 752–758 10.1038/nn1472 15895084

[B42] Delarasse C. Auger R. Gonnord P. Fontaine B. Kanellopoulos J. M. (2011). The purinergic receptor P2X7 triggers alpha-secretase-dependent processing of the amyloid precursor protein. J. Biol. Chem. 286, 2596–2606 10.1074/jbc.M110.200618 21081501 PMC3024755

[B43] de Rivero Vaccari J. P. Bastien D. Yurcisin G. Pineau I. Dietrich W. D. De Koninck Y. (2012). P2X4 receptors influence inflammasome activation after spinal cord injury. J. Neurosci. 32, 3058–3066 10.1523/JNEUROSCI.4930-11.2012 22378878 PMC6622016

[B44] Di Virgilio F. Ceruti S. Bramanti P. Abbracchio M. P. (2009). Purinergic signalling in inflammation of the central nervous system. Trends Neurosci. 32, 79–87 10.1016/j.tins.2008.11.003 19135728

[B45] Diaz-Hernandez J. I. Gomez-Villafuertes R. León-Otegui M. Hontecillas-Prieto L. Del Puerto A. Trejo J. L. (2012). *In vivo* P2X7 inhibition reduces amyloid plaques in Alzheimer's disease through GSK3β and secretases. Neurobiol. Aging 33, 1816–1828 10.1016/j.neurobiolaging.2011.09.040 22048123

[B46] Díaz-Hernández M. Díez-Zaera M. Sánchez-Nogueiro J. Gómez-Villafuertes R. Canals J. M. Alberch J. (2009). Altered P2X7-receptor level and function in mouse models of Huntington's disease and therapeutic efficacy of antagonist administration. FASEB J. 23, 1893–1906 10.1096/fj.08-122275 19171786

[B47] Domercq M. Perez-Samartin A. Aparicio D. Alberdi E. Pampliega O. Matute C. (2010). P2X7 receptors mediate ischemic damage to oligodendrocytes. Glia 58, 730–740 10.1002/glia.20958 20029962

[B48] Domercq M. Sánchez-Gómez M. V. Areso P. Matute C. (1999). Expression of glutamate transporters in rat optic nerve oligodendrocytes. Eur. J. Neurosci. 11, 2226–2236 10.1046/j.1460-9568.1999.00639.x 10383611

[B49] Domercq M. Sánchez-Gómez M. V. Sherwin C. Etxebarria E. Fern R. Matute C. (2007). System xc- and glutamate transporter inhibition mediates microglial toxicity to oligodendrocytes. J. Immunol. 178, 6549–6556 17475885 10.4049/jimmunol.178.10.6549

[B50] Dou Y. Wu H. J. Li H. Q. Qin S. Wang Y. E. Li J. (2012). Microglial migration mediated by ATP-induced ATP release from lysosomes. Cell Res. 22, 1022–1033 10.1038/cr.2012.10 22231629 PMC3367529

[B51] Engel T. Gomez-Villafuertes R. Tanaka K. Mesuret G. Sanz-Rodriguez A. Garcia-Huerta P. (2012). Seizure suppression and neuroprotection by targeting the purinergic P2X7 receptor during status epilepticus in mice. FASEB J. 26, 1616–1628 10.1096/fj.11-196089 22198387

[B52] Espey M. G. Kustova Y. Sei Y. Basile A. S. (1998). Extracellular glutamate levels are chronically elevated in the brains of LP-BM5-infected mice: a mechanism of retrovirus-induced encephalopathy. J. Neurochem. 71, 2079–2087 10.1046/j.1471-4159.1998.71052079.x 9798933

[B53] Eun S. Y. Hong Y. H. Kim E. H. Jeon H. Suh Y. H. Lee J. E. (2004). Glutamate receptor-mediated regulation of c-fos expression in cultured microglia. Biochem. Biophys. Res. Commun. 325, 320–327 10.1016/j.bbrc.2004.10.035 15522236

[B54] Fang K. M. Yang C. S. Sun S. H. Tzeng S. F. (2009). Microglial phagocytosis attenuated by short-term exposure to exogenous ATP through P2X receptor action. J. Neurochem. 111, 225–237 10.1111/j.1471-4159.2009.06409.x 19860838

[B55] Färber K. Markworth S. Pannasch U. Nolte C. Prinz V. Kronenberg G. (2008). The ectonucleotidase cd39/ENTPDase1 modulates purinergic-mediated microglial migration. Glia 56, 331–341 10.1002/glia.20606 18098126

[B56] Ferrari D. Villalba M. Chiozzi P. Falzoni S. Ricciardi-Castagnoli P. Di Virgilio F. (1996). Mouse microglial cells express a plasma membrane pore gated by extracellular ATP. J. Immunol. 156, 1531–1539 8568257

[B57] Ferrini F. Trang T. Mattioli T. A. Laffray S. Del'guidice T. Lorenzo L. E. (2013). Morphine hyperalgesia gated through microglia-mediated disruption of neuronal Cl(-) homeostasis. Nat. Neurosci. 16, 183–192 10.1038/nn.3295 23292683 PMC4974077

[B58] Fogal B. Li J. Lobner D. McCullough L. D. Hewett S. J. (2007). System x(c)- activity and astrocytes are necessary for interleukin-1 beta-mediated hypoxic neuronal injury. J. Neurosci. 27, 10094–10105 10.1523/JNEUROSCI.2459-07.2007 17881516 PMC6672668

[B59] Fontainhas A. M. Wang M. Liang K. J. Chen S. Mettu P. Damani M. (2011). Microglial morphology and dynamic behavior is regulated by ionotropic glutamatergic and GABAergic neurotransmission. PLoS ONE 6:e15973 10.1371/journal.pone.0015973 21283568 PMC3026789

[B60] Franke H. Günther A. Grosche J. Schmidt R. Rossner S. Reinhardt R. (2004). P2X7 receptor expression after ischemia in the cerebral cortex of rats. J. Neuropathol. Exp. Neurol. 63, 686–699 15290894 10.1093/jnen/63.7.686

[B61] Franke H. Schepper C. Illes P. Krügel U. (2007). Involvement of P2X and P2Y receptors in microglial activation *in vivo*. Purinergic Signal. 3, 435–445 10.1007/s11302-007-9082-y 18404456 PMC2072928

[B62] Fredholm B. B. Ijzerman A. P. Jacobson K. A. Klotz K. N. Linden J. (2001). International Union of Pharmacology. XXV. Nomenclature and classification of adenosine receptors. Pharmacol. Rev. 53, 527–552 11734617 PMC9389454

[B63] Gandelman M. Peluffo H. Beckman J. S. Cassina P. Barbeito L. (2010). Extracellular ATP and the P2X7 receptor in astrocyte-mediated motor neuron death: implications for amyotrophic lateral sclerosis. J. Neuroinflammation 7:33 10.1186/1742-2094-7-33 20534165 PMC2901222

[B64] Gao X. F. Wang W. Yu Q. Burnstock G. Xiang Z. H. He C. (2011). Astroglial P2X7 receptor current density increased following long-term exposure to rotenone. Purinergic Signal. 7, 65–72 10.1007/s11302-011-9218-y 21484098 PMC3083135

[B65] Gottlieb M. Matute C. (1997). Expression of ionotropic glutamate receptor subunits in glial cells of the hippocampal CA1 area following transient forebrain ischemia. J. Cereb. Blood Flow Metab. 17, 290–300 10.1097/00004647-199703000-00006 9119902

[B66] Guo L. H. Schluesener H. J. (2005). Lesional accumulation of P2X4 receptor-macrophages in rat CNS during experimental autoimmune encephalomyelitis. Neuroscience 134, 199–205 10.1016/j.neuroscience.2005.04.026 15964696

[B67] Hanisch U. K. Kettenmann H. (2007). Microglia: active sensor and versatile effector cells in the normal and pathologic brain. Nat. Neurosci. 10, 1387–1394 10.1038/nn1997 17965659

[B68] Hanley P. J. Kronlage M. Kirschning C. del Rey A. Di Virgilio F. Leipziger J. (2012). Transient P2X7 receptor activation triggers macrophage death independent of Toll-like receptors 2 and 4, caspase-1, and pannexin-1 proteins. J. Biol. Chem. 287, 10650–10663 10.1074/jbc.M111.332676 22235111 PMC3323034

[B69] Harada K. Hide I. Seki T. Tanaka S. Nakata Y. Sakai N. (2011). Extracellular ATP differentially modulates Toll-like receptor 4-mediated cell survival and death of microglia. J. Neurochem. 116, 1138–1147 10.1111/j.1471-4159.2011.07170.x 21210814

[B70] Harrigan T. J. Abdullaev I. F. Jourd'heuil D. Mongin A. A. (2008). Activation of microglia with zymosan promotes excitatory amino acid release via volume-regulated anion channels: the role of NADPH oxidases. J. Neurochem. 106, 2449–2462 10.1111/j.1471-4159.2008.05553.x 18624925 PMC2574595

[B71] Haselkorn M. L. Shellington D. K. Jackson E. K. Vagni V. A. Janesko-Feldman K. Dubey R. K. (2010). Adenosine A1 receptor activation as a brake on the microglial response after experimental traumatic brain injury in mice. J. Neurotrauma 27, 901–910 10.1089/neu.2009.1075 20121416 PMC2943944

[B72] Haskó G. Pacher P. Vizi E. S. Illes P. (2005). Adenosine receptor signaling in the brain immune system. Trends Pharmacol. Sci. 26, 511–516 10.1016/j.tips.2005.08.004 16125796 PMC2228262

[B73] Haynes S. E. Hollopeter G. Yang G. Kurpius D. Dailey M. E. Gan W. B. (2006). The P2Y12 receptor regulates microglial activation by extracellular nucleotides. Nat. Neurosci. 9, 1512–1519 10.1038/nn1805 17115040

[B74] Heyes M. P. Achim C. L. Wiley C. A. Major E. O. Saito K. Markey S. P. (1996). Human microglia convert l-tryptophan into the neurotoxin quinolinic acid. Biochem. J. 320, 595–597 8973572 10.1042/bj3200595 PMC1217971

[B75] Higashi Y. Segawa S. Matsuo T. Nakamura S. Kikkawa Y. Nishida K. (2011). Microglial zinc uptake via zinc transporters induces ATP release and the activation of microglia. Glia 59, 1933–1945 10.1002/glia.21235 22253048

[B76] Honda S. Sasaki Y. Ohsawa K. Imai Y. Nakamura Y. Inoue K. (2001). Extracellular ATP or ADP induce chemotaxis of cultured microglia through Gi/o-coupled P2Y receptors. J. Neurosci. 21, 1975–1982 11245682 10.1523/JNEUROSCI.21-06-01975.2001 PMC6762617

[B77] Honore P. Donnelly-Roberts D. Namovic M. Zhong C. Wade C. Chandran P. (2009). The antihyperalgesic activity of a selective P2X7 receptor antagonist, A-839977, is lost in IL-1alphabeta knockout mice. Behav. Brain Res. 204, 77–81 10.1016/j.bbr.2009.05.018 19464323

[B78] Idzko M. Hammad H. van Nimwegen M. Kool M. Willart M. A. Muskens F. (2007). Extracellular ATP triggers and maintains asthmatic airway inflammation by activating dendritic cells. Nat. Med. 13, 913–919 10.1038/nm1617 17632526

[B79] Jackman N. A. Uliasz T. F. Hewett J. A. Hewett S. J. (2010). Regulation of system x(c)(-)activity and expression in astrocytes by interleukin-1β: implications for hypoxic neuronal injury. Glia 58, 1806–1815 10.1002/glia.21050 20645408 PMC4451603

[B80] Jun D. J. Kim J. Jung S. Y. Song R. Noh J. H. Park Y. S. (2007). Extracellular ATP mediates necrotic cell swelling in SN4741 dopaminergic neurons through P2X7 receptors. J. Biol. Chem. 282, 37350–37358 10.1074/jbc.M707915200 17962183

[B81] Kaur C. Sivakumar V. Ang L. S. Sundaresan A. (2006). Hypoxic damage to the periventricular white matter in neonatal brain: role of vascular endothelial growth factor, nitric oxide and excitotoxicity. J. Neurochem. 98, 1200–1216 10.1111/j.1471-4159.2006.03964.x 16787408

[B82] Kawanokuchi J. Shimizu K. Nitta A. Yamada K. Mizuno T. Takeuchi H. (2008). Production and functions of IL-17 in microglia. J. Neuroimmunol. 194, 54–61 10.1016/j.jneuroim.2007.11.006 18164424

[B83] Kettenmann H. Hanisch U. K. Noda M. Verkhratsky A. (2011). Physiology of microglia. Physiol. Rev. 91, 461–553 10.1152/physrev.00011.2010 21527731

[B84] Khakh B. S. North R. A. (2012). Neuromodulation by extracellular ATP and P2X receptors in the CNS. Neuron 76, 51–69 10.1016/j.neuron.2012.09.024 23040806 PMC4064466

[B85] Kim B. Jeong H. K. Kim J. H. Lee S. Y. Jou I. Joe E. H. (2011). Uridine 5′-diphosphate induces chemokine expression in microglia and astrocytes through activation of the P2Y6 receptor. J. Immunol. 186, 3701–3709 10.4049/jimmunol.1000212 21317391

[B86] Kim H. J. Ajit D. Peterson T. S. Wang Y. Camden J. M. Gibson Wood W. (2012). Nucleotides released from Aβ_1–42_-treated microglial cells increase cell migration and Aβ_1–42_ uptake through P2Y_2_ receptor activation. J. Neurochem. 121, 228–238 10.1111/j.1471-4159.2012.07700.x 22353164 PMC3323761

[B87] Kim J. E. Kang T. C. (2011). The P2X7 receptor-pannexin-1 complex decreases muscarinic acetylcholine receptor-mediated seizure susceptibility in mice. J. Clin. Invest. 121, 2037–2047 10.1172/JCI44818 21505260 PMC3083785

[B88] Kim J. E. Kwak S. E. Jo S. M. Kang T. C. (2009). Blockade of P2X receptor prevents astroglial death in the dentate gyrus following pilocarpine-induced status epilepticus. Neurol. Res. 31, 982–988 10.1179/174313209X389811 19138473

[B89] Kim J. Y. Kanai Y. Chairoungdua A. Cha S. H. Matsuo H. Kim D. K. (2001). Human cystine/glutamate transporter: cDNA cloning and upregulation by oxidative stress in glioma cells. Biochim. Biophys. Acta 1512, 335–3344 10.1016/S0005-2736(01)00338-8 11406111

[B90] Kobayashi K. Yamanaka H. Fukuoka T. Dai Y. Obata K. Noguchi K. (2008). P2Y12 receptor upregulation in activated microglia is a gateway of p38 signaling and neuropathic pain. J. Neurosci. 28, 2892–2902 10.1523/JNEUROSCI.5589-07.2008 18337420 PMC6670682

[B91] Kobayashi K. Yamanaka H. Yanamoto F. Okubo M. Noguchi K. (2012). Multiple P2Y subtypes in spinal microglia are involved in neuropathic pain after peripheral nerve injury. Glia 60, 1529–1539 10.1002/glia.22373 22736439

[B92] Koizumi S. Ohsawa K. Inoue K. Kohsaka S. (2013). Purinergic receptors in microglia: functional modal shifts of microglia mediated by P2 and P1 receptors. Glia 61, 47–54 10.1002/glia.22358 22674620

[B93] Koizumi S. Shigemoto-Mogami Y. Nasu-Tada K. Shinozaki Y. Ohsawa K. Tsuda M. (2007). UDP acting at P2Y6 receptors is a mediator of microglial phagocytosis. Nature 446, 1091–1095 10.1038/nature05704 17410128 PMC3464483

[B94] Korn T. Magnus T. Jung S. (2005). Autoantigen specific T cells inhibit glutamate uptake in astrocytes by decreasing expression of astrocytic glutamate transporter GLAST: a mechanism mediated by tumor necrosis factor-alpha. FASEB J. 19, 1878–1880 10.1096/fj.05-3748fje 16123171

[B95] Koscsó B. Csóka B. Selmeczy Z. Himer L. Pacher P. Virág L. (2012). Adenosine augments IL-10 production by microglial cells through an A2B adenosine receptor-mediated process. J. Immunol. 188, 445–453 10.4049/jimmunol.1101224 22116830 PMC3384725

[B96] Kreutzberg G. W. (1996). Microglia: a sensor for pathological events in the CNS. Trends Neurosci. 19, 312–318 10.1016/0166-2236(96)10049-7 8843599

[B97] Kuehnel M. P. Reiss M. Anand P. K. Treede I. Holzerm D. Hoffmann E. (2009a). Sphingosine-1-phosphate receptors stimulate macrophage plasma-membrane actin assembly via ADP release, ATP synthesis and P2X7R activation. J. Cell Sci. 122, 505–512 10.1242/jcs.034207 19174470

[B98] Kuehnel M. P. Rybin V. Anand P. K. Anes E. Griffiths G. (2009b). Lipids regulate P2X7-receptor-dependent actin assembly by phagosomes via ADP translocation and ATP synthesis in the phagosome lumen. J. Cell Sci. 122, 499–504 10.1242/jcs.034199 19174471

[B99] Lawson L. J. Perry V. H. Dri P. Gordon S. (1990). Heterogeneity in the distribution and morphology of microglia in the normal adult mouse brain. Neuroscience 39, 151–170 10.1016/0306-4522(90)90229-W 2089275

[B100] Le Feuvre R. A. Brough D. Touzani O. Rothwell N. J. (2003). Role of P2X7 receptors in ischemic and excitotoxic brain injury *in vivo*. J. Cereb. Blood Flow Metab. 23, 381–384 12621313 10.1097/01.WCB.0000048519.34839.97

[B101] Lee H. G. Won S. M. Gwag B. J. Lee Y. B. (2011). Microglial P2X_7_ receptor expression is accompanied by neuronal damage in the cerebral cortex of the APPswe/PS1dE9 mouse model of Alzheimer's disease. Exp. Mol. Med. 43, 7–14 21088470 10.3858/emm.2011.43.1.001 PMC3041940

[B102] León-Otegui M. Gómez-Villafuertes R. Díaz-Hernández J. I. Díaz-Hernández M. Miras-Portugal M. T. Gualix J. (2011). Opposite effects of P2X7 and P2Y2 nucleotide receptors on α-secretase-dependent APP processing in Neuro-2a cells. FEBS Lett. 585, 2255–2262 10.1016/j.febslet.2011.05.048 21651910

[B103] Lerma J. (2003). Roles and rules of kainate receptors in synaptic transmission. Nat. Rev. Neurosci. 4, 481–495 10.1038/nrn1118 12778120

[B104] Li F. Wang L. Li J. W. Gong M. He L. Feng R. (2011). Hypoxia induced amoeboid microglial cell activation in postnatal rat brain is mediated by ATP receptor P2X4. BMC Neurosci. 12:111 10.1186/1471-2202-12-111 22053919 PMC3239293

[B105] Li J. Baud O. Vartanian T. Volpe J. J. Rosenberg P. A. (2005). Peroxynitrite generated by inducible nitric oxide synthase and NADPH oxidase mediates microglial toxicity to oligodendrocytes. Proc. Natl. Acad. Sci. U.S.A. 102, 9936–9941 10.1073/pnas.0502552102 15998743 PMC1174990

[B106] Li Y. Du X. F. Liu C. S. Wen Z. L. Du J. L. (2012). Reciprocal regulation between resting microglial dynamics and neuronal activity *in vivo*. Dev. Cell 23, 1189–1202 10.1016/j.devcel.2012.10.027 23201120

[B107] Liu G. J. Nagarajah R. Banati R. B. Bennett M. R. (2009). Glutamate induces directed chemotaxis of microglia. Eur. J. Neurosci. 29, 1108–1118 10.1111/j.1460-9568.2009.06659.x 19302147

[B108] Locovei S. Scemes E. Qiu F. Spray D. C. Dahl G. (2007). Pannexin1 is part of the pore forming unit of the P2X(7) receptor death complex. FEBS Lett. 581, 483–488 10.1016/j.febslet.2006.12.056 17240370 PMC1868681

[B109] Loram L. C. Harrison J. A. Sloane E. M. Hutchinson M. R. Sholar P. Taylor F. R. (2009). Enduring reversal of neuropathic pain by a single intrathecal injection of adenosine 2A receptor agonists: a novel therapy for neuropathic pain. J. Neurosci. 29, 14015–14025 10.1523/JNEUROSCI.3447-09.2009 19890011 PMC2799253

[B110] Lu Y. M. Tao R. R. Huang J. Y. Li L. T. Liao M. H. Li X. M. (2012). P2X(7) signaling promotes microsphere embolism-triggered microglia activation by maintaining elevation of Fas ligand. J. Neuroinflammation 9:172 10.1186/1742-2094-9-172 22789015 PMC3420259

[B111] Luongo L. Petrelli R. Gatta L. Giordano C. Guida F. Vita P. (2012). 5′-Chloro-5′-deoxy-(and#177;)-ENBA, a potent and selective adenosine A1 receptor agonist, alleviates neuropathic pain in mice through functional glial and microglial changes without affecting motor or cardiovascular functions. Molecules 17, 13712–13726 10.3390/molecules171213712 23174891 PMC6268894

[B112] Lynch M. A. (2009). The multifaceted profile of activated microglia. Mol. Neurobiol. 40, 139–156 10.1007/s12035-009-8077-9 19629762

[B113] Lyons S. A. Chung W. J. Weaver A. K. Ogunrinu T. Sontheimer H. (2007). Autocrine glutamate signaling promotes glioma cell invasion. Cancer Res. 67, 9463–9471 10.1158/0008-5472.CAN-07-2034 17909056 PMC2045073

[B114] MacVicar B. A. Thompson R. J. (2010). Non-junction functions of pannexin-1 channels. Trends Neurosci. 33, 93–102 10.1016/j.tins.2009.11.007 20022389

[B115] Maeda M. Tsuda M. Tozaki-Saitoh H. Inoue K. Kiyama H. (2010). Nerve injury-activated microglia engulf myelinated axons in a P2Y12 signaling-dependent manner in the dorsal horn. Glia 58, 1838–1846 10.1002/glia.21053 20665560

[B116] Manning S. M. Talos D. M. Zhou C. Selip D. B. Park H. K. Park C. J. (2008). NMDA receptor blockade with memantine attenuates white matter injury in a rat model of periventricular leukomalacia. J. Neurosci. 28, 6670–6678 10.1523/JNEUROSCI.1702-08.2008 18579741 PMC2800040

[B117] Matute C. Torre I. Pérez-Cerdá F. Pérez-Samartín A. Alberdi E. Etxebarria E. (2007). P2X(7) receptor blockade prevents ATP excitotoxicity in oligodendrocytes and ameliorates experimental autoimmune encephalomyelitis. J. Neurosci. 27, 9525–9533 10.1523/JNEUROSCI.0579-07.2007 17728465 PMC6673129

[B118] McLarnon J. G. Ryu J. K. Walker D. G. Choi H. B. (2006). Upregulated expression of purinergic P2X(7) receptor in Alzheimer disease and amyloid-beta peptide-treated microglia and in peptide-injected rat hippocampus. J. Neuropathol. Exp. Neurol. 65, 1090–1097 10.1097/01.jnen.0000240470.97295.d3 17086106

[B119] McLarnon J. G. Zhang L. Goghari V. Lee Y. B. Walz W. Krieger C. (1999). Effects of ATP and elevated K+ on K+ currents and intracellular Ca2+ in human microglia. Neuroscience 91, 343–352 10.1016/S0306-4522(98)00491-6 10336083

[B120] McMullan S. M. Phanavanh B. Li G. G. Barger S. W. (2012). Metabotropic glutamate receptors inhibit microglial glutamate release. ASN Neuro. [Epub ahead of print]. 10.1042/AN20120044 22770428 PMC3413012

[B121] Mead E. L. Mosley A. Eaton S. Dobson L. Heales S. J. Pocock J. M. (2012). Microglial neurotransmitter receptors trigger superoxide production in microglia; consequences for microglial-neuronal interactions. J. Neurochem. 121, 287–301 10.1111/j.1471-4159.2012.07659.x 22243365

[B122] Melani A. Amadio S. Gianfriddo M. Vannucchi M. G. Volontè C. Bernardi G. (2006). P2X7 receptor modulation on microglial cells and reduction of brain infarct caused by middle cerebral artery occlusion in rat. J. Cereb. Blood Flow Metab. 26, 974–982 10.1038/sj.jcbfm.9600250 16395292

[B123] Merrill J. E. Ignarro L. J. Sherman M. P. Melinek J. Lane T. E. (1993). Microglial cell cytotoxicity of oligodendrocytes is mediated through nitric oxide. J. Immunol. 151, 2132–2141 8102159

[B124] Miao E. A. Rajan J. V. Aderem A. (2011). Caspase-1-induced pyroptotic cell death. Immunol. Rev. 243, 206–214 10.1111/j.1600-065X.2011.01044.x 21884178 PMC3609431

[B125] Mills J. H. Kim D. G. Krenz A. Chen J. F. Bynoe M. S. (2012). A2A adenosine receptor signaling in lymphocytes and the central nervous system regulates inflammation during experimental autoimmune encephalomyelitis. J. Immunol. 188, 5713–5722 10.4049/jimmunol.1200545 22529293 PMC3358473

[B126] Monif M. Reid C. A. Powell K. L. Smart M. L. Williams D. A. (2009). The P2X7 receptor drives microglial activation and proliferation: a trophic role for P2X7R pore. J. Neurosci. 29, 3781–3791 10.1523/JNEUROSCI.5512-08.2009 19321774 PMC6665035

[B127] Murphy N. Cowley T. R. Richardson J. C. Virley D. Upton N. Walter D. (2012). The neuroprotective effect of a specific P2X7 receptor antagonist derives from its ability to inhibit assembly of the NLRP3 inflammasome in glial cells. Brain Pathol. 22, 295–306 10.1111/j.1750-3639.2011.00531.x 21933296 PMC8092963

[B128] Murugan M. Sivakumar V. Lu J. Ling E. A. Kaur C. (2011). Expression of N-methyl D-aspartate receptor subunits in amoeboid microglia mediates production of nitric oxide via NF-κ B signaling pathway and oligodendrocyte cell death in hypoxic postnatal rats. Glia 59, 521–539 10.1002/glia.21121 21319220

[B129] Neher J. J. Neniskyte U. Zhao J. W. Bal-Price A. Tolkovsky A. M. Brown G. C. (2011). Inhibition of microglial phagocytosis is sufficient to prevent inflammatory neuronal death. J. Immunol. 186, 4973–4983 10.4049/jimmunol.1003600 21402900

[B130] Nimmerjahn A. Kirchhoff F. Helmchen F. (2005). Resting microglial cells are highly dynamic surveillants of brain parenchyma *in vivo*. Science 308, 1314–1318 10.1126/science.1110647 15831717

[B131] Noda M. Nakanishi H. Nabekura J. Akaike N. (2000). AMPA-kainate subtypes of glutamate receptor in rat cerebral microglia. J. Neurosci. 20, 251–258 10627602 10.1523/JNEUROSCI.20-01-00251.2000 PMC6774119

[B132] Nörenberg W. Langosch J. M. Gebicke-Haerter P. J. Illes P. (1994). Characterization and possible function of adenosine 5′-triphosphate receptors in activated rat microglia. Br. J. Pharmacol. 111, 942–950 8019772 10.1111/j.1476-5381.1994.tb14830.x PMC1910099

[B133] North R. A. (2002). Molecular physiology of P2X receptors. Physiol. Rev. 82, 1013–1067 10.1152/physrev.00015.2002 12270951

[B134] Ochaion A. Bar-Yehuda S. Cohen S. Barer F. Patoka R. Amital H. (2009). The anti-inflammatory target A(3) adenosine receptor is over-expressed in rheumatoid arthritis, psoriasis and Crohn's disease. Cell. Immunol. 258, 115–122 10.1016/j.cellimm.2009.03.020 19426966

[B135] Ogunrinu T. A. Sontheimer H. (2010). Hypoxia increases the dependence of glioma cells on glutathione. J. Biol. Chem. 285, 37716–37724 10.1074/jbc.M110.161190 20858898 PMC2988376

[B136] Ohsawa K. Irino Y. Nakamura Y. Akazawa C. Inoue K. Kohsaka S. (2007). Involvement of P2X4 and P2Y12 receptors in ATP-induced microglial chemotaxis. Glia 55, 604–616 10.1002/glia.20489 17299767

[B137] Ohsawa K. Sanagi T. Nakamura Y. Suzuki E. Inoue K. Kohsaka S. (2012). Adenosine A3 receptor is involved in ADP-induced microglial process extension and migration. J. Neurochem. 121, 217–227 10.1111/j.1471-4159.2012.07693.x 22335470

[B138] Oka A. Belliveau M. J. Rosenberg P. A. Volpe J. J. (1993). Vulnerability of oligodendroglia to glutamate: pharmacology, mechanisms, and prevention. J. Neurosci. 13, 1441–1453 8096541 10.1523/JNEUROSCI.13-04-01441.1993 PMC6576718

[B139] Orr A. G. Orr A. L. Li X.-J. Gross R. E. Traynelis S. F. (2009). Adenosine A2A receptor mediates microglial process retraction. Nat. Neurosci. 12, 872–878 10.1038/nn.2341 19525944 PMC2712729

[B140] Oyanguren-Desez O. Rodríguez-Antigüedad A. Villoslada P. Domercq M. Alberdi E. Matute C. (2011). Gain-of-function of P2X7 receptor gene variants in multiple sclerosis. Cell Calcium 50, 468–472 10.1016/j.ceca.2011.08.002 21906809

[B141] Pampliega O. Domercq M. Soria F. N. Villoslada P. Rodríguez-Antigüedad A. Matute C. (2011). Increased expression of cystine/glutamate antiporter in multiple sclerosis. J. Neuroinflammation 8:63 10.1186/1742-2094-8-63 21639880 PMC3117706

[B142] Paolicelli R. C. Bolasco G. Pagani F. Maggi L. Scianni M. Panzanelli P. (2011). Synaptic pruning by microglia is necessary for normal brain development. Science 333, 1456–1458 10.1126/science.1202529 21778362

[B143] Parvathenani L. K. Tertyshnikova S. Greco C. R. Roberts S. B. Robertson B. Posmantur R. (2003). P2X7 mediates superoxide production in primary microglia and is up-regulated in a transgenic mouse model of Alzheimer's disease. J. Biol. Chem. 278, 13309–13317 10.1074/jbc.M209478200 12551918

[B144] Pascual O. Ben Achour S. Rostaing P. Triller A. Bessis A. (2012). Microglia activation triggers astrocyte-mediated modulation of excitatory neurotransmission. Proc. Natl. Acad. Sci. U.S.A. 109, 197–205 10.1073/pnas.1111098109 22167804 PMC3268269

[B145] Pedata F. Corsi C. Melani A. Bordoni F. Latini S. (2001). Adenosine extracellular brain concentrations and role of A2A receptors in ischemia. Ann. N.Y. Acad. Sci. 939, 74–84 10.1111/j.1749-6632.2001.tb03614.x 11462806

[B146] Pelegrin P. Surprenant A. (2006). Pannexin-1 mediates large pore formation and interleukin-1beta release by the ATP-gated P2X7 receptor. EMBO J. 25, 5071–5082 10.1038/sj.emboj.7601378 17036048 PMC1630421

[B147] Pellegatti P. Raffaghello L. Bianchi G. Piccardi F. Pistoia V. Di Virgilio F. (2008). Increased level of extracellular ATP at tumor sites: *in vivo* imaging with plasma membrane luciferase. PLoS ONE 3:2599 10.1371/journal.pone.0002599 18612415 PMC2440522

[B148] Peng W. Cotrina M. L. Han X. Yu H. Bekar L. Blum L. (2009). Systemic administration of an antagonist of the ATP-sensitive receptor P2X7 improves recovery after spinal cord injury. Proc. Natl. Acad. Sci. U.S.A. 106, 12489–12493 10.1073/pnas.0902531106 19666625 PMC2718350

[B149] Perea G. Navarrete M. Araque A. (2009). Tripartite synapses: astrocytes process and control synaptic information. Trends Neurosci. 32, 421–431 10.1016/j.tins.2009.05.001 19615761

[B150] Piani D. Spranger M. Frei K. Schaffner A. Fontana A. (1992). Macrophage-induced cytotoxicity of N-methyl-D-aspartate receptor positive neurons involves excitatory amino acids rather than reactive oxygen intermediates and cytokines. Eur. J. Immunol. 22, 2429–2436 10.1002/eji.1830220936 1355433

[B151] Pinteaux-Jones F. Sevastou I. G. Fry V. A. Heales S. Baker D. Pocock J. M. (2008). Myelin-induced microglial neurotoxicity can be controlled by microglial metabotropic glutamate receptors. J. Neurochem. 106, 442–454 10.1111/j.1471-4159.2008.05426.x 18419765

[B152] Pocock J. M. Kettenmann H. (2007). Neurotransmitter receptors on microglia. Trends Neurosci. 30, 527–735 10.1016/j.tins.2007.07.007 17904651

[B153] Ponomarev E. D. Maresz K. Tan Y. Dittel B. N. (2007). CNS-derived interleukin-4 is essential for the regulation of autoimmune inflammation and induces a state of alternative activation in microglial cells. J. Neurosci. 27, 10714–10721 10.1523/JNEUROSCI.1922-07.2007 17913905 PMC6672829

[B154] Popovich P. G. Tovar C. A. Wei P. Fisher L. Jakeman L. B. Basso D. M. (2011). A reassessment of a classic neuroprotective combination therapy for spinal cord injured rats: LPS/pregnenolone/indomethacin. Exp. Neurol. 233, 677–685 10.1016/j.expneurol.2011.11.045 22177997 PMC3477520

[B155] Qin S. Colin C. Hinners I. Gervais A. Cheret C. Mallat M. (2006). System Xc- and apolipoprotein E expressed by microglia have opposite effects on the neurotoxicity of amyloid-beta peptide 1–40. J. Neurosci. 26, 3345–3356 10.1523/JNEUROSCI.5186-05.2006 16554485 PMC6674113

[B156] Qin Z. Freitas E. Sullivan R. Mohan S. Bacelieri R. Branch D. (2010). Upregulation of xCT by KSHV-encoded microRNAs facilitates KSHV dissemination and persistence in an environment of oxidative stress. PLoS Pathog. 6:e1000742 10.1371/journal.ppat.1000742 20126446 PMC2813276

[B157] Ralevic V. Burnstock G. (1998). Receptors for purines and pyrimidines. Pharmacol. Rev. 50, 413–492 9755289

[B158] Rampe D. Wang L. Ringheim G. E. (2004). P2X7 receptor modulation of beta-amyloid- and LPS-induced cytokine secretion from human macrophages and microglia. J. Neuroimmunol. 147, 56–61 14741428 10.1016/j.jneuroim.2003.10.014

[B159] Ransohoff R. M. Perry V. H. (2009). Microglial physiology: unique stimuli, specialized responses. Annu. Rev. Immunol. 27, 119–145 10.1146/annurev.immunol.021908.132528 19302036

[B160] Rigato C. Buckinx R. Le-Corronc H. Rigo J. M. Legendre P. (2011). Pattern of invasion of the embryonic mouse spinal cord by microglial cells at the time of the onset of functional neuronal networks. Glia 59, 675–695 10.1002/glia.21140 21305616

[B161] Rigato C. Swinnen N. Buckinx R. Couillin I. Mangin J. M. Rigo J. M. (2012). Microglia proliferation is controlled by P2X7 receptors in a Pannexin-1-independent manner during early embryonic spinal cord invasion. J. Neurosci. 32, 11559–11573 10.1523/JNEUROSCI.1042-12.2012 22915101 PMC6703767

[B162] Rothstein J. D. (2002). Paving new pathways. Nat. Med. 8, 938–940 10.1038/nm0902-938 12205454

[B163] Santello M. Bezzi P. Volterra A. (2011). TNFα controls glutamatergic gliotransmission in the hippocampal dentate gyrus. Neuron 69, 988–1001 10.1016/j.neuron.2011.02.003 21382557

[B164] Santiago M. F. Veliskova J. Patel N. K. Lutz S. E. Caille D. Charollais A. (2011). Targeting pannexin1 improves seizure outcome. PLoS ONE 6:e25178 10.1371/journal.pone.0025178 21949881 PMC3175002

[B165] Sanz J. M. Chiozzi P. Ferrari D. Colaianna M. Idzko M. Falzoni S. (2009). Activation of microglia by amyloid {beta} requires P2X7 receptor expression. J. Immunol. 182, 4378–4385 10.4049/jimmunol.0803612 19299738

[B166] Sasaki Y. Hoshi M. Akazawa C. Nakamura Y. Tsuzuki H. Inoue K. (2003). Selective expression of Gi/o-coupled ATP receptor P2Y12 in microglia in rat brain. Glia 44, 242–250 10.1002/glia.10293 14603465

[B167] Schwab J. M. Guo L. Schluesener H. J. (2005). Spinal cord injury induces early and persistent lesional P2X4 receptor expression. J. Neuroimmunol. 163, 185–189 10.1016/j.jneuroim.2005.02.016 15885321

[B168] Schwartz M. Butovsky O. Brück W. Hanisch U. K. (2006). Microglial phenotype: is the commitment reversible? Trends Neurosci. 29, 68–74 10.1016/j.tins.2005.12.005 16406093

[B169] Schwarzschild M. A. Agnati L. Fuxe K. Chen J. F. Morelli M. (2006). Targeting adenosine A2A receptors in Parkinson's disease. Trends Neurosci. 29, 647–654 10.1016/j.tins.2006.09.004 17030429

[B170] Sieger D. Moritz C. Ziegenhals T. Prykhozhij S. Peri F. (2012). Long-range Ca2+ waves transmit brain-damage signals to microglia. Dev. Cell 22, 1138–1148 10.1016/j.devcel.2012.04.012 22632801

[B171] Sierra A. Encinas J. M. Deudero J. J. Chancey J. H. Enikolopov G. Overstreet-Wadiche L. S. (2010). Microglia shape adult hippocampal neurogenesis through apoptosis-coupled phagocytosis. Cell Stem Cell 7, 483–495 10.1016/j.stem.2010.08.014 20887954 PMC4008496

[B172] Silverman W. R. de Rivero Vaccari J. P. Locovei S. Qiu F. Carlsson S. K. Scemes E. (2009). The pannexin 1 channel activates the inflammasome in neurons and astrocytes. J. Biol. Chem. 284, 18143–18151 10.1074/jbc.M109.004804 19416975 PMC2709345

[B173] Stellwagen D. Malenka R. C. (2006). Synaptic scaling mediated by glial TNF-alpha. Nature 440, 1054–1059 10.1038/nature04671 16547515

[B174] Streit W. J. Morioka T. Kalehua A. N. (1992). MK-801 prevents microglial reaction in rat hippocampus after forebrain ischemia. Neuroreport 3, 146–148 1535800 10.1097/00001756-199202000-00006

[B175] Surprenant A. Rassendren F. Kawashima E. North R. A. Buell G. (1996). The cytolytic P2Z receptor for extracellular ATP identified as a P2X receptor. Science 272, 735–738 10.1126/science.272.5262.735 8614837

[B176] Suzuki T. Hide I. Ido K. Kohsaka S. Inoue K. Nakata Y. (2004). Production and release of neuroprotective tumor necrosis factor by P2X7 receptor-activated microglia. J. Neurosci. 24, 1–7 10.1523/JNEUROSCI.3792-03.2004 14715932 PMC6729576

[B177] Swanson C. J. Bures M. Johnson M. P. Linden A. M. Monn J. A. Schoepp D. D. (2005). Metabotropic glutamate receptors as novel targets for anxiety and stress disorders. Nat. Rev. Drug Discov. 4, 131–144 10.1038/nrd1630 15665858

[B178] Taguchi K. Tamba M. Bannai S. Sato H. (2007). Induction of cystine/glutamate transporter in bacterial lipopolysaccharide induced endotoxemia in mice. J. Inflamm. 4:20 10.1186/1476-9255-4-20 17897437 PMC2039726

[B179] Tahraoui S. L. Marret S. Bodénant C. Leroux P. Dommergues M. A. Evrard P. (2001). Central role of microglia in neonatal excitotoxic lesions of the murine periventricular white matter. Brain Pathol. 11, 56–71 11145204 10.1111/j.1750-3639.2001.tb00381.x PMC8098534

[B180] Takano T. Lin J. H. Arcuino G. Gao Q. Yang J. Nedergaard M. (2001). Glutamate release promotes growth of malignant gliomas. Nat. Med. 7, 1010–1015 10.1038/nm0901-1010 11533703

[B181] Taylor D. L. Diemel L. T. Cuzner M. L. Pocock J. M. (2002). Activation of group II metabotropic glutamate receptors underlies microglial reactivity and neurotoxicity following stimulation with chromogranin A, a peptide up-regulated in Alzheimer's disease. J. Neurochem. 82, 1179–1191 10.1046/j.1471-4159.2002.01062.x 12358765

[B182] Taylor D. L. Diemel L. T. Pocock J. M. (2003). Activation of microglial group III metabotropic glutamate receptors protects neurons against microglial neurotoxicity. J. Neurosci. 23, 2150–2160 12657674 10.1523/JNEUROSCI.23-06-02150.2003 PMC6742009

[B183] Taylor D. L. Jones F. Kubota E. S. Pocock J. M. (2005). Stimulation of microglial metabotropic glutamate receptor mGlu2 triggers tumor necrosis factor alpha-induced neurotoxicity in concert with microglial-derived Fas ligand. J. Neurosci. 25, 2952–2964 10.1523/JNEUROSCI.4456-04.2005 15772355 PMC6725132

[B184] Thomas D. M. Kuhn D. M. (2005). MK-801 and dextromethorphan block microglial activation and protect against methamphetamine-induced neuro-toxicity. Brain Res. 1050, 190–198 10.1016/j.brainres.2005.05.049 15987631

[B185] Thompson R. J. Jackson M. F. Olah M. E. Rungta R. L. Hines D. J. Beazely M. A. (2008). Activation of pannexin-1 hemichannels augments aberrant bursting in the hippocampus. Science 322, 1555–1559 10.1126/science.1165209 19056988

[B186] Thompson R. J. Zhou N. MacVicar B. A. (2006). Ischemia opens neuronal gap junction hemichannels. Science 312, 924–927 10.1126/science.1126241 16690868

[B187] Toyomitsu E. Tsuda M. Yamashita T. Tozaki-Saitoh H. Tanaka Y. Inoue K. (2012). CCL2 promotes P2X4 receptor trafficking to the cell surface of microglia. Purinergic Signal. 8, 301–310 10.1007/s11302-011-9288-x 22222817 PMC3350584

[B188] Tozaki-Saitoh H. Tsuda M. Miyata H. Ueda K. Kohsaka S. Inoue K. (2008). P2Y12 receptors in spinal microglia are required for neuropathic pain after peripheral nerve injury. J. Neurosci. 28, 4949–4956 10.1523/JNEUROSCI.0323-08.2008 18463248 PMC6670742

[B189] Tran M. D. Neary J. T. (2006). Purinergic signaling induces thrombospondin-1 expression in astrocytes. Proc. Natl. Acad. Sci. U.S.A. 103, 9321–9326 10.1073/pnas.0603146103 16754856 PMC1482608

[B190] Tremblay M. È. (2011). The role of microglia at synapses in the healthy CNS: novel insights from recent imaging studies. Neuron Glia Biol. 7, 67–76 10.1017/S1740925X12000038 22418067

[B191] Tremblay M. È. Lowery R. L. Majewska A. K. (2010). Microglial interactions with synapses are modulated by visual experience. PLoS Biol. 8:e1000527 10.1371/journal.pbio.1000527 21072242 PMC2970556

[B192] Tsuda M. Beggs S. Salter M. W. Inoue K. (2013). Microglia and intractable chronic pain. Glia 61, 55–61 10.1002/glia.22379 22740331

[B193] Tsuda M. Masuda T. Kitano J. Shimoyama H. Tozaki-Saitoh H. Inoue K. (2009). IFN-gamma receptor signaling mediates spinal microglia activation driving neuropathic pain. Proc. Natl. Acad. Sci. U.S.A. 106, 8032–8037 10.1073/pnas.0810420106 19380717 PMC2683100

[B194] Tsuda M. Tozaki-Saitoh H. Masuda T. Toyomitsu E. Tezuka T. Yamamoto T. (2008). Lyn tyrosine kinase is required for P2X(4) receptor upregulation and neuropathic pain after peripheral nerve injury. Glia 56, 50–58 10.1002/glia.20591 17918263

[B195] Tsutsui S. Schnermann J. Noorbakhsh F. Henry S. Yong V. W. Winston B. W. (2004). A1 adenosine receptor upregulation and activation attenuates neuroinflammation and demyelination in a model of multiple sclerosis. J. Neurosci. 24, 1521–1529 10.1523/JNEUROSCI.4271-03.2004 14960625 PMC6730323

[B196] Ulmann L. Hatcher J. P. Hughes J. P. Chaumont S. Green P. J. Conquet F. (2008). Up-regulation of P2X4 receptors in spinal microglia after peripheral nerve injury mediates BDNF release and neuropathic pain. J. Neurosci. 28, 11263–11268 10.1523/JNEUROSCI.2308-08.2008 18971468 PMC6671487

[B197] Vinet J. Weering H. R. Heinrich A. Kälin R. E. Wegner A. Brouwer N. (2012). Neuroprotective function for ramified microglia in hippocampal excitotoxicity. J. Neuroinflammation 9, 27 10.1186/1742-2094-9-27 22293457 PMC3292937

[B198] Volonte C. Apolloni S. Skaper S. D. Burnstock G. (2012). P2X7 receptors: channels, pores and more. CNS Neurol. Disord. Drug Targets 11, 705–721 10.2174/187152712803581137 22963440

[B199] Von Lubitz D. K. Lin R. C. Popik P. Carter M. F. Jacobson K. A. (1994). Adenosine A3 receptor stimulation and cerebral ischemia. Eur. J. Pharmacol. 263, 59–67 10.1016/0014-2999(94)90523-1 7821362 PMC3426360

[B200] Von Lubitz D. K. Simpson K. L. Lin R. C. (2001). Right thing at a wrong time? Adenosine A3 receptors and cerebroprotection in stroke. Ann. N.Y. Acad. Sci. 939, 85–96 10.1111/j.1749-6632.2001.tb03615.x 11462807

[B201] Wake H. Moorhouse A. J. Jinno S. Kohsaka S. Nabekura J. (2009). Resting microglia directly monitor the functional state of synapses *in vivo* and determine the fate of ischemic terminals. J. Neurosci. 29, 3974–3980 10.1523/JNEUROSCI.4363-08.2009 19339593 PMC6665392

[B202] Wake H. Moorhouse A. J. Miyamoto A. Nabekura J. (2013). Microglia: actively surveying and shaping neuronal circuit structure and function. Trends Neurosci. 36, 209–217 10.1016/j.tins.2012.11.007 23260014

[B203] Walz W. Ilschner S. Ohlemeyer C. Banati R. Kettenmann H. (1993). Extracellular ATP activates a cation conductance and a K+ conductance in cultured microglial cells from mouse brain. J. Neurosci. 13, 4403–4411 7692013 10.1523/JNEUROSCI.13-10-04403.1993 PMC6576379

[B204] Wang X. Arcuino G. Takano T. Lin J. Peng W. G. Wan P. (2004). P2X7 receptor inhibition improves recovery after spinal cord injury. Nat. Med. 10, 821–827 10.1038/nm1082 15258577

[B205] Weisman G. A. Camden J. M. Peterson T. S. Ajit D. Woods L. T. Erb L. (2012). P2 receptors for extracellular nucleotides in the central nervous system: role of P2X7 and P2Y2 receptor interactions in neuroinflammation. Mol. Neurobiol. 46, 96–113 10.1007/s12035-012-8263-z 22467178 PMC3439567

[B206] Witting A. Walter L. Wacker J. Möller T. Stella N. (2004). P2X7 receptors control 2-arachidonoylglycerol production by microglial cells. Proc. Natl. Acad. Sci. U.S.A. 101, 3214–3219 10.1073/pnas.0306707101 14976257 PMC365769

[B207a] Witting A. Chen L. Cudaback E. Straiker A. Walter L. Rickman B. (2006). Experimental autoimmune encephalomyelitis disrupts endocannabinoid-mediated neuroprotection. Proc. Natl. Acad. Sci. U.S.A. 103, 6362–6367 10.1073/pnas.0510418103 16571660 PMC1458883

[B207] Wu L. J. Zhuo M. (2008). Resting microglial motility is independent of synaptic plasticity in mammalian brain. J. Neurophysiol. 99, 2026–2032 10.1152/jn.01210.2007 18256162

[B208] Wu S. Z. Bodles A. M. Porter M. M. Griffin W. S. Basile A. S. Barger S. W. (2004). Induction of serine racemase expression and D-serine release from microglia by amyloid beta-peptide. J. Neuroinflammation 1:2 10.1186/1742-2094-1-2 15285800 PMC483052

[B209] Xiang Z. Burnstock G. (2005). Expression of P2X receptors on rat microglial cells during early development. Glia 52, 119–126 10.1002/glia.20227 15920729

[B210] Yao S. Q. Li Z. Z. Huang Q. Y. Li F. Wang Z. W. Augusto E. (2012). Genetic inactivation of the adenosine A(2A) receptor exacerbates brain damage in mice with experimental autoimmune encephalomyelitis. J. Neurochem. 123, 100–112 10.1111/j.1471-4159.2012.07807.x 22639925

[B211] Ye Z. C. Rothstein J. D. Sontheimer H. (1999). Compromised glutamate transport in human glioma cells: reduction-mislocalization of sodium-dependent glutamate transporters and enhanced activity of cystine-glutamate exchange. J. Neurosci. 19, 10767–10777 10594060 10.1523/JNEUROSCI.19-24-10767.1999 PMC6784962

[B212] Ye Z. C. Sontheimer H. (1999). Glioma cells release excitotoxic concentrations of glutamate. Cancer Res. 59, 4383–4391 10485487

[B213] Yiangou Y. Facer P. Durrenberger P. Chessell I. P. Naylor A. Bountra C. (2006). COX-2, CB2 and P2X7-immunoreactivities are increased in activated microglial cells/macrophages of multiple sclerosis and amyotrophic lateral sclerosis spinal cord. BMC Neurol. 6:12 10.1186/1471-2377-6-12 16512913 PMC1413551

[B214] Zeng Y. Li Y. Chen R. S. He X. Yang L. Li W. (2010). Overexpression of xCT induces up-regulation of 14-3-3beta in Kaposi's sarcoma. Biosci. Rep. 30, 277–283 10.1042/BSR20090163 20100173 PMC2860696

[B215] Zhang Z. Zhang Z. Artelt M. Burnet M. Schluesener H. J. (2007). Dexamethasone attenuates early expression of three molecules associated with microglia/macrophages activation following rat traumatic brain injury. Acta Neuropathol. 113, 675–682 10.1007/s00401-007-0195-8 17265048

[B216] Zheng H. Zhu W. Zhao H. Wang X. Wang W. Li Z. (2010). Kainic acid-activated microglia mediate increased excitability of rat hippocampal neurons *in vitro* and *in vivo*: crucial role of interleukin-1beta. Neuroimmunomodulation 17, 31–38 10.1159/000243083 19816055

[B217] Zhu W. Zheng H. Shao X. Wang W. Yao Q. Li Z. (2010). Excitotoxicity of TNFalpha derived from KA activated microglia on hippocampal neurons *in vitro* and *in vivo*. J. Neurochem. 114, 386–396 10.1111/j.1471-4159.2010.06763.x 20438614

