# Erratum: Neurotransmitter signaling in the pathophysiology of microglia

**DOI:** 10.3389/fncel.2013.00107

**Published:** 2013-07-09

**Authors:** María Domercq, Nuria Vazquez-Villoldo, Carlos Matute

**Affiliations:** Department of Neuroscience, University of the Basque CountryLeioa, Spain

At the top of **page 5**, the following changes need to be made.

… regulators of P2X4R expression in microglia have been described, such as the chemokine CCL2 (also known as monocyte chemoattractant protein, MCP-1; Biber et al., 2011; Toyomitsu et al., 2012),….

Leaving the corrected text as follows:

… regulators of P2X4R expression in microglia have been described, such as the chemokine CCL21 (Biber et al., 2011)….

**Page 16**. The reference by Toyomitsu et al., 2012 should be removed.
